# New Molecular Phylogenetic Evidence Confirms Independent Origin of Coxal Combs in the Families of the ‘Cydnoid’ Complex (Hemiptera: Heteroptera: Pentatomoidea)

**DOI:** 10.3390/insects15100792

**Published:** 2024-10-11

**Authors:** Jerzy A. Lis, Paweł J. Domagała, Barbara Lis

**Affiliations:** Institute of Biology, University of Opole, Oleska 22, 45-052 Opole, Poland; pdomagala@uni.opole.pl (P.J.D.); canta@uni.opole.pl (B.L.)

**Keywords:** Cydnidae, Parastrachiidae, Thaumastellidae, Thyreocoridae, burrower bugs, morphology, phylogeny, 16S rDNA

## Abstract

**Simple Summary:**

Four families (Cydnidae, Parastrachiidae, Thaumastellidae, and Thyreocoridae) in the superfamily Pentatomoidea are morphologically defined by a similar body outline and the presence of a series of stout setae on the distal margin of the coxae. These structures, called coxal combs, are thought to protect the coxal–trochanteral articulation from damage caused by soil and sand particles and have not been observed in other true bugs (Hemiptera: Heteroptera). Therefore, they have been consistently considered a synapomorphy for these four families. However, their independent origin in these families has been suggested by nuclear ribosomal DNA sequence analysis (28S rDNA and 18S rDNA). This study investigated whether the analysis of mitochondrial 16S ribosomal DNA sequences would confirm these results. In addition, we examined whether any group of species possessing the coxal combs could be considered a well-supported independent monophylum.

**Abstract:**

Coxal combs, found only in members of the ‘cydnoid’ complex (comprising four families: Cydnidae, Parastrachiidae, Thaumastellidae, and Thyreocoridae) within the superfamily Pentatomoidea, have long been regarded as a character confirming their close evolutionary relationship. However, many studies have demonstrated that these four families are phylogenetically distant. Others have been treated as subfamilies of the broadly defined Cydnidae, with the coxal combs as the only character linking them. This is the first study on the origin of coxal combs in species of all families and subfamilies that represent the broadly conceived Cydnidae (69 species in 39 genera). Moreover, this study presents the first 16S rDNA gene sequences providing a basis for such analyses. The analyses included DNA sequences of 62 species in 34 genera of Cydnidae *sensu stricto*, three species in two genera of Thyreocoridae, two species in two genera of Parastrachiidae, and two species in one genus of Thaumastellidae. The sequence analysis in the family Cydnidae covered 35 species representing 19 genera of the subfamily Cydninae, 16 species in eight genera of the subfamily Sehirinae, five species in two genera of Amnestinae, three species in three genera of Garsauriinae, two species in one genus of Cephalocteinae, and one species of Amaurocorinae. The results of our study demonstrate the independent origin of coxal combs in taxa of the ‘cydnoid’ complex within the superfamily Pentatomoidea. They confirm the polyphyly not only of the entire ‘cydnoid’ complex but also of the family Cydnidae itself.

## 1. Introduction

Some insects have evolved morphological adaptations that facilitate their burrowing activities. They are adapted to spending at least part of their lives underground and have developed a suite of morphological adaptations for subterranean existence [1,2,3,4]. The most significant and prevalent adaptation is the modification of the anterior legs, often referred to as ‘fossorial legs’. These legs have undergone specific morphological changes that allow them to dig effectively into the ground [1,2,3,4,5,6].

In insects, the typical anterior leg comprises five segments: the coxa, trochanter, femur, tibia, and tarsus [1,4]. However, the primary morphological adaptations associated with digging are modifications to the tibiae, which exhibit distinctive structural changes [1,2,3,4,5,6]. Furthermore, fossorial insects frequently have somewhat stout setae arranged in rows on various body parts, including the leg segments, suggesting an adaptation for digging [7,8,9]. Despite the morphological similarity of the fossorial forelegs in representatives of various insect orders, including Coleoptera, Hemiptera, and Orthoptera, these modifications are classic examples of convergence among insects [1,2,3,4].

Within Hemiptera, the fossorial anterior legs are observed in nymphs of the families Cicadidae and Tettigarctidae (Cicadomorpha: Cicadoidea), for example [2,10,11,12,13], and in nymphs and adults of the family Cydnidae (Heteroptera: Pentatomomorpha: Pentatomoidea) [3,5,6,14,15].

Although the coxa—the first segment of the insect leg—is often its smallest part, it may also play an essential role in the fossorial function of the anterior leg [3,4,9].

In Heteroptera, the coxae are characterised by significant morphological diversity, with varying degrees of hair and setal cover [3,4,9,16,17]. However, the presence of various types of setae and hairs, which are found in groups close to the trochanteral margins, is not a common occurrence [9,16,17].

In some species of Nepomorpha (Corixidae, Ochteridae, and Gelastocoridae), the coxae are externally covered with hairs and have small groupings of these near the trochanter [17]. A comparable arrangement of the coxal setae attached to the trochanter, known as the coxal combs, has been identified in several families of the superfamily Pentatomoidea within the infraorder Pentatomomorpha [5,6,9,14,15,16]. However, these groups of setae in Nepomorpha were proposed to be part of the stridulatory structure [17], whereas in Pentatomoidea, their proposed function was the prevention of dust particles entering the coxal-trochanteral articulation [14,18] or the cleaning of soil particles from the antennae [19,20]. Nevertheless, as previously suggested [9,14,16,21], all these functions in Pentatomoidea appear to be connected to the fossorial habits of its members, which possess coxal combs.

Nevertheless, recent findings have also revealed that these coxal combs are present in pentatomoid species that reside on plants and do not engage in burrowing activities [9,16]. It is noteworthy that a comparable type of scale-like setae, which constitute the coxal combs in numerous burrowing bugs, was also identified at the apex of the first antennomeres and the apex of the first labial segment in species belonging to the family Thaumastellidae [22].

**Figure 1:**
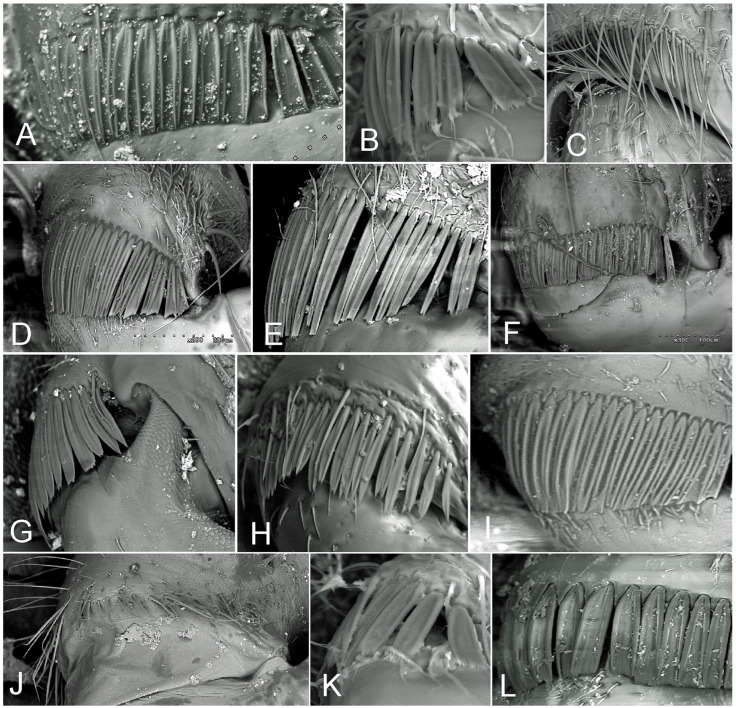
Coxal combs in representatives of the ‘cydnoid complex’. (**A**–**I**) Cydnidae. (**J**) Parastrachiidae. (**K**) Thaumastellidae. (**L**) Thyreocoridae. (**A**) Amaurocorinae: *Linospa candida*. (**B**) Amnestinae: *Amnestus pusillus*. (**C**) Cephalocteinae: *Scaptocoris australis*. (**D**) Cydninae: *Cydnus pericarti*. (**E**) Cydninae: *Lactistes vericulatus*. (**F**) Garsauriinae: *Blaena setosa*. (**G**) Garsauriinae: *Garsauria usambarica*. (**H**) Sehirinae: *Sehirus luctuosus*. (**I**) Sehirinae: *Ochetostethomorpha secunda*. (**J**) *Parastrachia nagaensis*. (**K**) *Thaumastella aradoides*. (**L**) *Galgupha vinculata*. The coxal combs depicted in the photographs are not presented at the same scale.

Most notably, these specific coxal structures attached to the trochanter, called coxal combs (Figure 1), are found only in representatives of four pentatomoid families (Cydnidae, Parastrachiidae, Thaumastellidae, and Thyreocoridae) and are unknown in other Heteroptera families [6,9,14,15,20,21,23]. Therefore, they have long been considered the only character confirming the close evolutionary relationship of these four families [6,9,14,15,20,21,23].

Regardless of the reliability of coxal combs as a phylogenetic criterion, two independent concepts of classification of these four families have been used to date. The first concept considers Parastrachiidae, Thaumastellidae, Thyreocoridae, and Cydnidae to be separate families [5,9,16,24,25,26,27,28,29], and the second considers all these families to be subfamilies of the broadly defined Cydnidae [6,14,21,30,31].

However, many recent studies based on morphology and DNA sequences [9,15,16,25,28,29] have shown the phylogenetic distinctiveness of these four families and challenged their grouping into one clade. Nevertheless, in a recent monograph on the true bugs of the world [6], an essential treatise for everyone studying Heteroptera, the classification combining Cydnidae, Parastrachiidae, Thaumastellidae, and Thyreocoridae into a single-family taxon (Cydnidae *sensu* Schuh & Weirauch) has been applied again.

Schuh & Weirauch [6] identified over a dozen morphological features to define the broadly described Cydnidae; of these, only the coxal combs in species of all four families are still sometimes considered the most crucial for grouping them into one taxon [9,15,19,22,25,30,31,32,33].

To date, molecular studies including all four families of the ‘cydnoid’ complex (Cydnidae *sensu* Schuh & Weirauch [6]) have predominately used nuclear 18S rDNA and 28S rDNA gene sequences and occasionally mitochondrial 16S rDNA [15,16,28,29,34,35,36]. Moreover, in some papers that considered the superfamily Pentatomoidea, several genes other than those mentioned above were included in the analyses (e.g., cytochrome oxidase I, cytochrome oxidase II, 12S rDNA, *Hox* genes, and the complete mitogenomes) [15,34,35,36,37,38,39,40,41,42,43,44].

Nevertheless, all phylogenomic studies to date have only included species belonging to two subfamilies: Sehirinae and Cydninae. Species of the other four subfamilies (Amaurocorinae, Amnestinae, Cephalocteinae, and Garsauriinae) have never been used in such investigations. Therefore, we chose one additional molecular marker, the mitochondrial 16S rDNA gene, to ascertain whether the species exhibiting coxal combs are phylogenetically related. Moreover, these gene sequences can also be obtained from ancient museum specimens [45,46,47,48,49,50,51], which allowed us to obtain DNA sequences of many more species compared with previous studies. We analysed as many species of the ‘cydnoid’ complex (Cydnidae *sensu* Schuh & Weirauch, 2020) as possible using taxa representing all of its families and subfamilies for the first time.

## 2. Materials and Methods

### 2.1. Selection of Taxa

This study selected 111 terminal taxa for analysis, with 106 species in the ingroup (Pentatomomorpha) and five species in the outgroup (Cimicomorpha: Nabidae, Miridae, and Tingidae) (Table S1). Within the ingroup, 93 species represented the subfamily Penta-tomoidea, including 69 species of the ‘cydnoid’ complex (two species in two genera of Parastrachiidae, three species in two genera of Thyreocoridae, two species in one genus of Thaumastellidae, and 62 species in 34 genera of Cydnidae). Newly obtained sequences for 62 species (59 in Cydnidae, one in Pentatomidae, one in Thyreocoridae, and one in Nabidae) were deposited into GenBank (Table S2).

### 2.2. DNA Extraction

Most specimens used for DNA extraction were dried individuals preserved in the first author’s collection (Table S2). However, some ethanol-preserved samples collected by the authors or other researchers were also included in the analysis (the ethanol-preserved specimens used in this study were the same as those listed in Lis & Domagała) [29]. Total genomic DNA was isolated from thoracic muscle tissue for each species using a Sherlock AX DNA Extraction Kit (A&A Biotechnology, Gdańsk, Poland) according to the manufacturer’s recommended protocol, irrespective of the form of sample preservation.

### 2.3. PCR Amplification, Purification, and Sequencing

The PCR amplifications were performed in a volume of 50 μL using the ready-to-use mixture PCR Mix Plus (A&A Biotechnology, Gdańsk, Poland) and a set of primers: F: LR-J-12961, R: LR-J-13417 [52,53]. The PCR mixture contained 25 μL of PCR Mix Plus, 2 μL of template DNA, 1 μL of each primer (10 μM), and ultrapure water to a final volume of 50 μL. The PCR reaction was performed according to the protocol described by Lis et al. [39,49]. Amplification products were analysed on a 2% agarose gel with the addition of 5 µL of SimplySafe stain (EURx, Gdańsk, Poland) and visualised under UV light. The PCR products were purified using the Clean-Up Kit (A&A Biotechnology, Gdańsk, Poland) and sequenced bidirectionally (Genomed S.A., Warszawa, Poland). The sequences obtained were verified by BLAST searches and deposited into the NCBI GenBank under the accession numbers PP357091–PP357152 (Table S2). After DNA extraction, the remains of the specimens were placed in tubes containing 96% ethanol and stored in a deep freezer at the Institute of Biology, University of Opole, Poland (see Table S2 for University of Opole voucher numbers). All the newly sequenced specimens were identified to species by the first author (JAL).

### 2.4. Phylogenetic Analysis

The input alignment dataset for the phylogenetic analyses was generated using MAFFT with Auto strategy [54]. Maximum likelihood (ML) and Bayesian (BI) analyses were performed on the dataset (File S1) to test the common evolutionary ancestry of coxal combs in the ‘cydnoid’ complex of families (i.e., in Cydnidae *sensu* Schuh & Weirauch). The best-fitting substitution model was tested using ModelFinder in IQ-TREE [55] for the ML analysis and jModelTest2 [56] for the BI analysis. The resulting models (GTR+F+I+G4 for ML; GTR+I+G for BI) were used for the phylogenetic analyses. The ML tree was generated via IQ-TREE [55] on the web server [57] using 10,000 replicates and the ultrafast bootstrap method [58]. Bayesian inference analysis was performed with MrBayes 3.2.6 [59] using XSEDE on the CIPRES server [60]. Two independent runs with four chains were carried out for 10 million generations, with the Markov chain Monte Carlo analysis sampled every 1000 generations, and a 25% burn-in was discarded. Convergence was determined by checking that the final mean standard deviation of the split frequencies was <0.05 and that each parameter’s potential scale reduction factor was close to 1.

**Figure 2:**
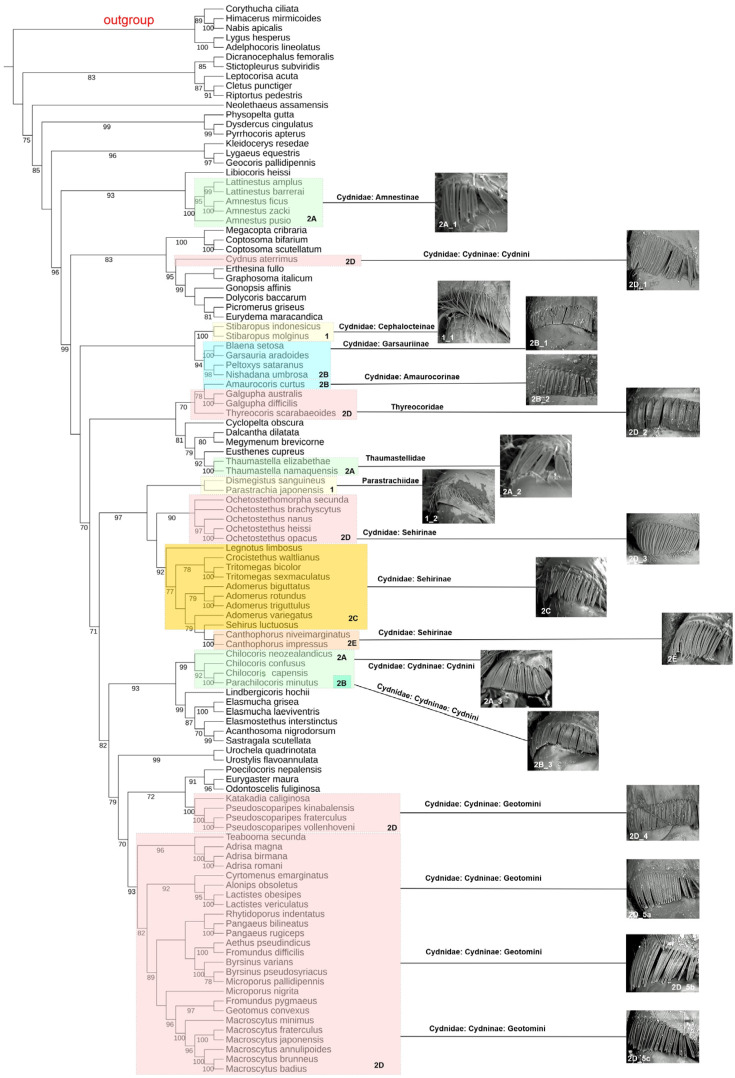
Phylogenetic consensus tree of the ‘cydnoid’ complex of families (Cydnidae, Parastrachiidae, Thaumastellidae, Thyreocoridae) based on a maximum likelihood analysis of the *16S* rDNA. Node labels—Ultrafast Bootstrap values (see Section 2.4), the Bootstrap values ≥70% are shown next to the branches. The type of coxal comb is depicted for each monophyletic group within the analysed taxa (**1**—coxal combs composed of an irregular row of long, stout, apically sharpened setae; **2A**–**E**—coxal combs composed of a regular row of scale-like setae). (**1_1**) Cydnidae: Cephalocteinae: *Scaptocoris australis*. (**1_2**) Parastrachiidae: *Parastrachia japonensis*. (**2A_1**) Amnestinae: *Amnestus pusillus*. (**2A_2**) Thaumastellidae: *Thaumastella aradoides*. (**2A_3**) Cydnidae: Cydninae: *Chilocoris nitidus*, 1st coxa. (**2B_1**) Cydnidae: Garsauriinae: *Blaena setosa*. (**2B_2**) Cydnidae: Amaurocorinae: *Linospa candida*. (**2B_3**) Cydnidae: Cydninae: *Chilocoris nitidus*, 2nd coxa. (**2C**) Cydnidae: Sehirinae: *Sehirus luctuosus*. (**2D_1**) Cydnidae: Cydninae: *Cydnus aterrimus*. (**2D_2**) Thyreocoridae: *Galgupha vinculata*. (**2D_3**) Cydnidae: Sehiri-nae: *Ochetostethomorpha secunda*. (**2D_4**) Cydnidae: Cydninae: *Pseudostibaropus kinabalensis*. (**2D_5a**) Cydnidae: Cydninae: *Adrisa magna*. (**2D_5b**) Cydnidae: Cydninae: *Lactistes vericulatus*. (**2D_5c**) Cydnidae: Cydninae: *Macroscytus brunneus*. (**2E**) Cydnidae: Sehirinae: *Canthophorus impressus*.

The resulting consensus trees for both analyses (Figure S1) were visualised and edited using the online tool iTOL v5 [61] and prepared for publication using CorelDRAW 21. Clades were considered to be well-supported if they had bootstrap values of ≥90% and posterior probabilities of ≥0.95. The ML consensus tree was selected to present our study’s results (Figure 2).

### 2.5. Photographic Documentation

A Hitachi S-3000N microscope was used for scanning electron microscopy for the micrographs presented in Figure 1 and Figure 2. The following species were analysed: Cydnidae: Amaurocorinae: *Amaurocoris curtus* (Brullé, 1838); *Linospa candida* Horváth, 1889; Amnestinae: *Amnestus pusillus* Uhler, 1876; Cephalocteinae: Scaptocorini: *Scaptocoris australis* J.A. Lis, 1999; Cydninae: Cydnini: *Cydnus aterrimus* (Forster, 1771); *C*. *pericarti* J.A. Lis, 1996; *Chilocoris nitidus* Mayr, 1865; Cydninae: Geotomini *s. lato*: *Adrisa magna* (Uhler, 1861); *Lactistes veri-culatus* Schiødte, 1848; *Macroscytus brunneus* (Fabricius, 1803); *Pseudoscoparipes kinabalensis* J.A. Lis, 1994; Garsauriinae: *Blaena setosa* Walker, 1868; *Garsauria usambarica* Schouteden, 1905; Sehirinae: Sehirini *s. lato*: *Canthophorus impressus* (Horváth, 1881); *Sehirus luctuosus* Mulsant et Rey, 1866; *Ochetostethomorpha secunda* J.A. Lis & B. Lis, 2014. Parastrachiidae: *Parastrachia nagaensis* Distant, 1908; *P. japonensis* (Scott, 1880). Thauma-stellidae: *Thaumastella aradoides* Horváth, 1896. Thyreocoridae: *Galgupha vinculata* (Germar, 1839).

### 2.6. Morphological Data

Following the methods of Lis [9], two main types and several subtypes of coxal combs were recognised. The first type is characterised by the coxal comb composed of an irregular row of long, stout, apically sharpened setae. The second type differs significantly from the first by having more or less broad, scale-like, or gutter-like setae instead of the customarily developed long, stout setae. Within this group, five distinct subtypes can be identified, which are characterised by setal form and arrangement variations. They have been extensively delineated by Lis [9], and thus, their descriptions are not reiterated herein.

## 3. Results

### 3.1. Sequence Alignment

The final 16S rDNA alignment (File S1) contained 111 sequences with 486 sites. The numbers of conserved and variable sites were 117 and 336, respectively. A total of 287 sites were parsimony-informative, and 44 were singletons. The average nucleotide frequencies (in per cent) were 42.8 (T), 32.0 (A), 16.4 (G), and 8.8 (C).

### 3.2. Tree Topology

Both analyses (ML and BI) resulted in consensus trees with similar topologies (see Figure S1 for the ML and BI tree). The differences in clade placement and the values of the reliability coefficients (bootstrap in ML; posterior probability in BI) were minor (Figure 2 and Figure S1). Most importantly, both trees indicated the independent origin of coxal combs within taxa classified as belonging to the ‘cydnoid’ complex (Cydnidae *sensu* Schuh & Weirauch [6] (Figure 2 and Figure S1), thus supporting the polyphyly of Cydnidae (regardless of family classification).

The results of our study confirm previous findings concerning the taxa included in the ‘cydnoid’ complex (Cydnidae *sensu* Schuh & Weirauch [6]). Both analyses support the separation of Thaumastellidae from other ‘cydnoid’ complex families and the polyphyly of the tribe Geotomini (Cydnidae: Cydninae). The BI analysis indicated that the second tribe of the subfamily Cydninae—Cydnini—is a monophylum. However, the ML analysis identified *Cydnus aterrimus* as not belonging to the clade containing other genera of Cydnini (i.e., species of *Chilocoris* and *Parachilocoris*), suggesting the polyphyly of the tribe.

Furthermore, four subfamilies (Amnestinae, Amaurocorinae, Cephalocteinae, and Garsauriinae) within the family Cydnidae *sensu* Pluot-Sigwalt & Lis [25] were shown to be separate from the subfamily Cydninae. Cephalocteinae was only considered a sister of Garsauriinae in the ML analysis. Notably, Amaurocoriinae was grouped with Thyreocoridae in both analyses.

The monophyly of the group containing the species of the subfamily Sehirinae (Cydnidae) and the family Parastrachiidae was confirmed in both analyses.

### 3.3. Types of Coxal Combs versus Classification of the Family Cydnidae

This study identified the following types of coxal combs among the taxa of the ‘cydnoid’ complex of families, mostly corresponding to the types and subtypes already proposed [9]. These are the coxal comb consisting of an irregular row of long, stout, apically sharpened setae (Type 1) or more or less broad, scale-like setae (Type 2, with five subtypes).

Most importantly, we found no correlations between the types of coxal combs and the monophyletic groups that were recovered during our analysis (Figure 2). Furthermore, we observed no correspondence between the structure of coxal combs and the current classification of the family Cydnidae (*sensu* Pluot-Sigwalt & Lis [25]) and the ‘cydnoid’ complex (Cydnidae *sensu* Schuh & Weirauch [9]). All this points to an independent origin of coxal combs within this ‘cydnoid’ group of families in the superfamily Pentatomoidea. Therefore, for the broadly defined Cydnidae (including Cydnidae *sensu stricto*, Parastrachiidae, Thaumastellidae, and Thyreocoridae), this character should no longer be considered a synapomorphy (Figure 2).

## 4. Discussion

### 4.1. Natural History of the Broadly Defined Cydnidae versus the Presence of Coxal Combs

Although all species belonging to the four families of pentatomoid insects (Cydnidae, Parastrachiidae, Thaumastellidae, and Thyreocoridae) are distinguished by the presence of coxal combs, which are typically associated with burrowing activities [9,14,16,19,20,21,30], some of them do not engage in typical fossorial behaviours [9,16]. Nevertheless, representatives of each family (and subfamily) within the broadly defined Cydnidae demonstrate apparent environmental relationships with the soil, sand, litter, or debris during their life cycle.

The species of Thaumastellidae reside in the ground or underground in small chambers or cavities situated beneath stones [18,22,62]. However, these species will venture from their designated shelters in search of seeds on the ground on which to feed [22].

In contrast, members of the family Parastrachiidae reside on plants, which serve as their hosts. For instance, *Parastrachia japonensis* inhabits and feeds on *Schepfia jasminodora* S. et Z. (Oleaceae) [18,30,63,64,65]. However, during the late summer and winter months, males and females leave the plants, descending to the ground for concealment, preferring crevices in leaf litter and locations beneath dead branches of the host plant [63,64,65].

The family Thyreocoridae predominantly comprises above-ground dweller species that feed on plants [5,18,66,67,68]. However, some members of this family burrow in the ground [18,68] or inhabit detritus [69], loose sand around plant roots [70], and sand steppes and dunes [66,71].

However, the most remarkable diversity of species associated with subterranean ecosystems is observed within the family Cydnidae.

Representatives of the subfamily Amaurocorinae (i.e., species from its three genera, *Amaurocoris* Stål, *Angra* Schumacher, and *Linospa* Signoret) populate the soil beneath plants and stones in sandy habitats [72,73,74].

Among the subfamily Cydninae, whose biological characteristics have been the subject of intense scientific investigation, most species have been observed to feed on plant roots [5,18,69,74,75,76,77,78,79,80,81,82,83]. However, some are cavernicolous (cave-dwelling) and live in bat guano [72,84,85], whereas others have been closely associated with ants and can be uncovered in anthills [86,87,88].

The tribes represented by species of the subfamily Cephalocteinae, specifically Cephalocteini and Scaptocorini, also display soil-digging behaviours. These species exhibit adaptations to the underground environment that are highly specific and well-developed [69,74,79,89,90,91,92]. For example, *Cephalocteus scarabaeoides* (Fabricius), a species belonging to the tribe Cephalocteini, has adapted to digging in sandy habitats, including marine beaches and dunes, at depths reaching 60 cm [91,92,93,94]. Furthermore, the species of the tribe Scaptocorini are well-known for their ability to live and reproduce in subterranean environments. Those of the genus *Scaptocoris* Perty can burrow considerably at the nymphal and adult stages [5,95,96,97,98,99,100,101], and move by scraping the soil with their scythe-like anterior tibiae [5,96,97,98,99,100,101]. They are primarily sap-feeders, deriving nutrients from the roots of host plants [5,96,97,98,99,100,101]. Similarly, species of *Stibaropus* Dallas and *Schiodtella* Signoret, at both adult and nymphal stages, reside in the soil beneath plants (occasionally reaching depths of up to 50 cm) and feed on the roots of their hosts [69,74,75,79,90,102].

Although the Amnestinae species are not strongly fossorial, they have been sampled under stones and debris [5,6,23,103,104,105,106,107,108] and in litter in a humid forest [104,109]. Some *Amnestus* species have been observed feeding directly on the seeds of various plants, e.g., *Amnestus ficus* Mayorga & Cervantes on *Ficus* L. (Moraceae) [104]. Furthermore, nymphs and adults have been discovered at depths approximately 10 cm below the ground’s surface [104]. Adults and nymphs of *A. subferrugineus* (Westwood) have been observed feeding on seeds in guano within bat caves [110].

Notably, there are two subfamilies within the family Cydnidae, specifically the Garsauriinae and Sehirinae, whose species are entirely unrelated to the soil environment and have little to no ability to live or develop underground.

It has been suggested that species of the Garsauriinae (particularly those belonging to the genera *Garsauria* Walker and *Garsauriella* Linnavuori) may be mycetophagous, inhabiting the loose bark of fallen trees in rainforests [18,72,74,82,111]. Nevertheless, adult and nymphal specimens belonging to numerous species of *Blaena* Walker (the Australian genus of Garsauriinae) have been collected in terrestrial habitats, including soil, beach dunes, desert sand, and under rocks [112,113], suggesting their ability to excavate subterranean environments.

Members of the subfamily Sehirinae typically occupy, feed on, and reproduce on above-ground plant parts exclusively e.g., [6,18,23,27,71,72,74,79,82]. However, the species biology within this subfamily cannot be generalised, as there are known species whose biology differs from the accepted conventional rules. For example, females of certain species of Sehirinae (predominantly those specimens belonging to the *Sehirus* Amyot et Serville) spend a portion of their life cycle on the ground, where they deposit eggs in shallow cavities [5,6,18,114,115,116,117]. Furthermore, adults in this subfamily have been observed in detritus near and in their host plant roots [18,69,79,93,94]. Moreover, species of two closely related Sehirinae genera, *Ochetostethus* Fieber and *Ochetostethomorpha* Schumacher, have been collected regularly in sandy substrates, on the ground beneath diverse vegetation, or have been extracted from litter samples collected from beneath trees [18,69,72,94,118,119,120].

Based on the above data, most species of the broadly defined Cydnidae come into contact with various types of substrates (sand, soil, guano, fragments of litter, debris, etc.) at some point in their life cycle. On these substrates, individuals move, hide, or sometimes burrow deeply. This behaviour may result in the substrate particles obstructing or even blocking their ability to move at the junction of the coxa and trochanter. Therefore, it is likely that coxal combs, as bristles or setae, evolved on the posterior edge of the coxa to protect the coxa-trochanter junction from potential damage caused by contact with the diverse types of substrata.

Moreover, the data demonstrate that disparate types of coxal combs are observed in taxa that are closely related, whereas the same kinds of combs are present in unrelated taxa. It is, therefore, reasonable to assume that the origin of such structures on the coxa represents an adaptive solution reflecting similar responses to environmental pressures. Whether this is an example of a convergence phenomenon, as observed in the fossorial legs of various insect groups [1,2,3,4], or a parallel evolution [121] remains unanswered and requires further research.

### 4.2. Polyphyly of the Broadly Defined Cydnidae (Sensu Schuh & Weirauch, 2020)

Our molecular analysis does not support the previous morphology-based grouping of four families, namely Cydnidae, Parastrachiidae, Thaumastellidae, and Thyreocoridae, classified under Cydnidae *sensu* Schuh & Weirauch [6,14,15,18,20,21,31,122]. It is also noteworthy that all previous molecular studies, including analyses of nuclear and mitochondrial DNA gene sequences [15,16,29,34,35,36,37,38,39,40,41,42,43,44,45,46,47,48,49,50,51,52,53,54,55,56,57,58,59,60,61,62,63,64,65,66,123], have demonstrated that the broadly defined Cydnidae [6] is polyphyletic. Furthermore, comparative analyses of secondary and tertiary ribosomal RNA structures [28,29,34] have demonstrated that taxa regarded as subfamilies in the broadly defined Cydnidae (i.e., Amaurocorinae, Amnestinae, Cephalocteinae, Cydninae, Garsauriinae, Parastrachiinae, Sehiriniae, Thaumastellinae, and Thyreocorinae) cannot be grouped according to any of the characteristics given by Schuh and Weirauch [6].

### 4.3. Polyphyly of the Family Cydnidae Sensu Pluot-Sigwalt & Lis, 2008

Most molecular papers that have considered the family Cydnidae *sensu* Pluot-Sigwalt and Lis [25] have included only the taxa representing the subfamily Cydninae [15,34,37,38,40,42,43,44,124,125,126,127] or two subfamilies, namely, Cydninae and Sehirinae [16,28,35,39,41,128].

These studies were based on a limited number of species (typically two or three) and often included only a single species of the subfamily family Cydninae; therefore, it was challenging to ascertain the polyphyletic origin of the family. Notwithstanding these limitations, the findings of several studies [15,16,28,35] indicate a non-monophyletic origin for certain groups of burrower bug taxa, particularly within the subfamily Cydninae.

The only molecular study in which the DNA sequence analyses were based on data obtained from dozens of species representing all six subfamilies (Amaurocorinae, Amnestinae, Cephalocteinae, Cydninae, Garsauriinae, and Sehirinae), rather than data from a few species belonging only to the Cydninae and Sehirinae, is a study by Lis and Domagała [29]. The results obtained from analyses of secondary and tertiary 18S rRNA structures stood in marked contrast to the current classification of the family Cydnidae *sensu* Pluot-Sigwalt & Lis [25].

The results of this molecular study agree with those of previous studies, which have not only indicated that the broadly defined Cydnidae [6] is polyphyletic but also that the Cydnidae *sensu* Pluot-Sigwalt and Lis [25] should be considered to have a non-monophyletic origin [15,16,25,28,29,34,35,36,122]. Furthermore, our findings explicitly indicate that the classification of the family Cydnidae *sensu* Pluot-Sigwalt and Lis [25] should be reconsidered. A paper on the reclassification of this group of pentatomoid bugs, the necessity of which was recognised over 20 years ago [129], is currently in preparation.

### 4.4. Monophyly of Sehirinae + Parastrachiidae

To date, in all molecular analyses including species of both the subfamily Sehrinae and the family Parastrachiidae [16,29,35,36], they have consistently formed a highly supported clade distinct from all other representatives included in the family Cydnidae (regardless of the concept of its internal classification). Our study also revealed a highly supported clade containing two Parastrachiidae and sixteen Sehirinae species (Figure 2 and Figure S1). Thus, it seems reasonable to consider the group comprising the species of Sehirinae and Parastrachiidae as a well-documented monophylum.

Nevertheless, further confirmation is required before accepting the results of molecular studies based on the analysis of nuclear [16,28,29] and mitochondrial DNA (this study) encoding ribosomal RNAs. This is because the morphological structures of the taxa belonging to these two groups have yet to be thoroughly investigated.

## 5. Conclusions

This is the first study to investigate the origin of coxal combs in representatives of all families and subfamilies that represent the broadly conceived Cydnidae. The first 16S rDNA gene sequences provided a basis for these analyses. Our study identified the types of coxal combs among the taxa of the ‘cydnoid’ complex of families, mainly corresponding to the types and subtypes already proposed. No correlations were identified between the types of coxal combs and the monophyletic groups recovered during the analysis. Our results support the hypothesis that the coxal combs of taxa within the ‘cydnoid’ complex originated independently. Furthermore, our findings confirm the polyphyly of the entire ‘cydnoid’ complex of families and the family Cydnidae itself. For the broadly defined Cydnidae (including Cydnidae *sensu stricto*, Parastrachiidae, Thaumastellidae, and Thyreocoridae), this character should no longer be considered a synapomorphy. The distinctiveness of the family Thaumastellidae compared with other families within the ‘cydnoid’ complex was confirmed. The results also indicate the polyphyly of the tribe Geotomini (Cydnidae: Cydninae). Our analyses confirmed the monophyly of the group containing species of the subfamily Sehirinae (Cydnidae) and the family Parastrachiidae. Furthermore, our results suggest that this group is separate from all other representatives included in the family Cydnidae (regardless of the concept of its internal classification). It is acknowledged that the outcomes of analyses based on 16S rDNA gene sequences may not be adequate to ascertain phylogenetic relationships within the ‘cydnoid’ complex, despite the extensive number of species utilised. Furthermore, the need for Supplementary Data, such as genome-scale information, is recognised as a means to more accurately address the polyphyly observed in the ‘cydnoid’ complex and to facilitate an enhanced understanding of the origin of coxal combs.

## Data Availability

The original contributions presented in the study are included in the article and Supplementary Materials, further inquiries can be directed to the corresponding author.

## Supplementary Materials

The following supporting information can be downloaded at: https://www.mdpi.com/article/10.3390/insects15100792/s1, Figure S1: Consensus trees for the ML (left) and BI (right) phylogenetic analyses. File S1. The input alignment dataset for the phylogenetic analyses generated using MAFFT. Table S1: List of specimens used in the phylogenetic analysis, their geographic origin, GenBank accession numbers, and the sources for the sequences downloaded from GenBank. Table S2: List of specimens used for extraction and amplification during this study, their geographic origin, GenBank accession numbers, and University of Opole sample numbers.
