# Supplementary material for: New Molecular Phylogenetic Evidence Confirms Independent Origin of Coxal Combs in the Families of the ‘Cydnoid’ Complex (Hemiptera: Heteroptera: Pentatomoidea)
Source: Insects. 2024 Oct 11;15(10):792. doi: 10.3390/insects15100792 (PMC11509079; doi:10.3390/insects15100792)
Supplement: Supplementary file 1 [file insects-15-00792-s001.zip › File S1.pdf]

>Adelphocoris lineolatus
------------TGTTTTAAATAAAAGATTTAACCTGCTCTATGATT-------------
ATTAAATGGCTGCAGTATTTTGACTGTACAAAGGTAGCATAATAATTTGTCTTTTAATTG
AAGACTGGTATGAATGGTTGTATGAGAAATATACTTTCTTTATTTTATA---TA-AAAAA
ATTGATTTTTTAGTTAAAAAGCTAAAATATTT-T-TATAGGACGAGAAGACCCTATAGAA
CTTTACTTTAATA-TAAA------------TATA---CTATTTA----TATATT------
----------TTTAAAATT-TTA--TATTTTTATTAAAGTTTTGTTGGGGTGACAAAAAA
AAT-TATATAACTTTTTTTATT-AA----------ATCATTAATT-----TATGT----T
-TTT---TTGATCCTTTA-----T-----TATGGAAAAT--AAGATTAAGTTACCTTAGG
GATAAC
>Lygus hesperus
------------TTGTTTATATAAAAGATCTAACCTGCCCTATGATTT----------AT
TTTAAATGGCTGCAGTATTTTGACTGTACAAAGGTAGCATAATCATTTGTCTTTTAATTG
AAGGCTGGTATGAATGGTTGGATGAAAAATATACTTTCTTTATTTTATA---AA-AATAA
ATTAACTTTTTAGTCAAAAGGCTAAGATTTTA-T-TGTAGGACGAGAAGACCCTATAGAA
CTTTATTTTCTTT-TTATA--------TA--------ACTTTTT----TAGTTT------
----------TTAATAAGT-TTA--TATATTTATGTTAATTATGTTGGGGTGACAGAAAA
AAT-AATATAACTTTTTCTATT------TTTT----TCATTTATT-----TATGT----G
-TTT---TTGATCCTATA-----T-----TATGGATAAT--AAGATTAAGTTACCTTAGG
GATAAC
>Cletus punctiger
----------TAGATTTTTAATTTTAAGTCTAATCTGCCCGGTGATTA------------
ATTAAACGGCCGCAGTATTCTGACTGTGCGAAGGTAGCATAATCGTTTGTCTCTTAATTG
GGGGCTTGTATGAATGATTGGACGAGGGACTGACTTTTTTTATTTAATT--TTT-ATGAA
TTTGATTTTTGAGTTAAAAAGCTTAAATTTTT-T-TGCAAGACGAGAAGACCCTATAGAA
TTTTATTTTTAAT-TAAT------------TACTTA-CTATT------TGGTTT------
----------ATTAACATT-AGG--TAGTTTTTTAAGAATTTTGTGGGGGCGATATTGAA
ATT-AAAATAACTTTCATTTAT-AA---TTTT----TCATTATTT-----AATGT----A
TTTT---TTGATCCTTTA-----T-----TATGGATTAT--TAGATTAAATTACCTTAGG
GATAAC
>Leptocorisa acuta
-----------TAGTATTTTATTATAGGTCTAACCTGCCCAATGATTA----------AA
TTTGAATGGCCGCAGTATTTTGACTGTGCAAAGGTAGCATAATCATTTGTCTTTTAATTG
AAGGCTTGTATGAATGGTTGGACGAGGAACTAGCTTTCTTTAATTTAAT-ATTT-ATTAA
TTTAATTTTTAAGTCAAAAAGCTTAAATATTT-T-TGCAAGACGAGAAGACCCTATAGAA
TTTTATTTTATGG-TGAA------------GGTT---TTATATT----TAGTTT------
----------ATATTTATA-TAA--TTTTTATTATAAAATTTTGTTGGGGTGACATTGAG
ATT-TTATTAACTCTCAATTAT-TT---AATA----TCACTTATA-----AGTGT----A
TATT---TTGATCCTTTA-----T-----TATAGATTAT--AAGATTAAATTACCTTAGG
GATAAC
>Dicranocephalus femoralis
----------TAGATATTATATTTTAGGTCTGACCTGCCCTATGATTT----------TT
ATTAAATGGCTGCAGTATTTTAACTGTACAAAGGTAGCATAATCATTTGTCTTTTAATTG
GGGGCTAGAATGAATGGTTGGACGAGGGATGTACTTTCTTTATTTAAAA--TAA-ATGAA
TTTAATTTTTTAGTCAAAAAGCTTAAATTTTT-T-TGTTAGACGAGAAGACCCTATAGAA
TTTTATTTTATTA-TTAA------------TGCTTG-TTATTAG--GTTAAATT------
----------TGAATTTGT-AGG--AATTGGTAATAAAATTTTGTTGGGGTGACAGTGAA
ATT-TAAATAACTTTCATTAAT-TT---TATT----TCATTGATT-----AATGT----T
-TTA---TTGATCCTATA-----T-----TATGGATTAT--AAGATTAAATTACCTTAGG
GATAAC
>Riptortus pedestris
---------TAGATTTAATAATTTTAGGTCTAGTCTGCCCAATGATTT------------
ATTTAATGGCTGCAGTATTTTAACTGTACGAAGGTAGCATAATCATTTGTCTTTTAATTG
GAGGCTTGTATGAATGGCTGGACGAGGAACTTACTTTTTTTATTTAAAT--TTT-ATGAA

TTTAATTTTTTAGTCAAAAAGCTAAAATTTTT-T-TGCAAGACGAGAAGACCCTATAGAA
TTTTATTTATTTA-TATT------------AATTA--AATATTA----AGTTAT------
----------TTAACTATT-TTA--TTTATATAAATAAATTTTGTTGGGGTGACAGTGAA
ATT-TAAATAACTTTCATATAT-----ACAAT----TCATTGATT-----TATGT----A
-AAA---TTGATCCCTTT-----TT---ATTAGGATTAC--TAGATTAAATTACCTTAGG
GATAAC
>Kleidocerys resedae
----------TAGATTATTAATTTTAGGTCTATCCTGCTCAGTGATTT----------AT
TTTAAATAGCCGCAGTATTTTGACTGTGCAAAGGTAGCATAATCATTTGTCTTTTAATTG
AAGGCTTGTATGAATGGTTGGACGAGATATTAACTTTCTTTATTTAATT--TTT-ATGAA
TTTAATTTTTTAGTGAAAAAGCTTAAATTATT-T-TGTGGGACGAGAAGACCCTATAGAA
TTTAAGATTAATT-TATTA--------TA--------TTTAATT----AATTAT------
----------TTGATTATT-TTA--TATTATTCTTAATTTTTGGTTGGGGTGATTATAAA
ATT-TTCTTAACTTTTATATTT-----TATTT----TCATTGATT-----AGTGT----T
TTTA---TTGATCCTTTA-----T-----TAAGGATTAT--TAGATTAAATTACCTTAGG
GATAAC
>Lygaeus equestris
-----------TAGATATTAATTTTAGGTTTAATCTGCTCAGTGATTA----------AT
TTTAAATAGCTGCAGTATTTTGACTGTACAAAGGTAGCATAATCATTTGTCTTTTAATTG
GAGGCTTGTATGAATGATTGGACGAAAAATAATCTTTCTTTATTTAATA--ATT-TTGAA
TTTAATTTTTTAGTTAAAAAGCTAAAATGAAT-T-TGTAAGACGAGAAGACCCTATAGAA
TTTTATTTTATTT-ATTT------------TATTTA-TTTTATT----------------
----------TTTAAATGT-TTT--TAATTTAAATTAAATTTGGTTGGGGTGACTATGAA
AAT-TTTTTAACTTTCATTTAT-----TTTTT----TCATAAATT-----AATGT---TG
TATA---TTGATCCTGTT-----T-----TATAGATTAA--AAGATTAAATTACCTTAGG
GATAAC
>Neolethaeus assamensis
---------TAGATTTATTAATTTTAGGTCTGACCTGCCCAATGATGG---------AAA
GTTTAATGGCCGCAGTATTTTAACTGTGCAAAGGTAGCATAATCATTTGTCTCTTAATTG
GAGGCTTGTATGAAAGGTTGGACGAGAGATATTCTTTCTTTATTTAATA--TAA-ATTAA
TTTGATTTTTCAGTTAAAAAGCTGAGATCTTT-T-TGTGGGACGAGAAGACCCTATAGAA
TTTTATTATTTAT-TCTT------------ATTTAT-TTTATTT----TTGTTT------
----------TTAATATAT-TAC--TTAATTATTAATAATTTGGTTGGGGTGATTGTGAG
ATT-AAATTAACTCTTATTTAC-TT---TTTG----TCATTAATT-----AGTGT----T
-TAT---TTGATCCTTTG-----T-----TATGGATTTC--AAGATTAAATTACCTTAGG
GATAAC
>Stictopleurus subviridis
-----------TTGATTTTAATATAAGGTCGGGCCTGCCCAATGATTT-----------T
ATTTAATGGCCGCAGTATCCTAACTGTGCAAAGGTAGCATAATCATTTGTCTTTTAATTA
AAGGCTGGAATGAATGGTTGGACGAAGGATTAACTTTTTTTATTTAAAT--TTT-ATGAA
TTTAATTTTTTGGTTAAAAAGCTAAAATTTTT-T-TGTTAGACGAGAAGACCCTATAGAA
TTTTAATATTTAT-ATTT------------ATTTA--GTTATTT----TGATTT------
----------TGATAAAAT-TAG--TTATTTATAATTATTTTTGTTGGGGTGACAATGAA
AAT-TAAATAACTTTCATTATT-TT---TTAT----TCATTAATT-----TATGT----A
TATT---TTGATCCTATA-----T-----TATGGATTGT--AAGATTAAATTACCTTAGG
GATAAC
>Geocoris pallidipennis
----------TTGTTATATAATATTAGGTCTATCCTGCTCAGTGATTT------------
ATTAAATAGCCGCAGTATTTTGACTGTGCAAAGGTAGCATAATCATTTGTTTTTTAATTG
AAAACTTGTATGAATGGGTGGACGAGGAATTAACTTTCTTTATTAAAAT--TAAGTTTAA
TTTAATTTTTTAGTTAAAAAGCTAAAATTTAT-A-TTTAAGACGAGAAGACCCTATAGAA
TTTTATTAATCTT-TTTA------------CATCTA------------AAATTAATT---
----------TTTGTTTTT-AGA--TGTAAAAAGATTAATTTGGTTGGGGTGACTATGAA
ATT-AAAATAACTTTCATTATT-TA---TATT----TCATTGATT-----TATGT----T

-TTT---AAGATCCTGTA-----T-----TATGGATTAT--AAGATTAAATTACCTTAGG
GATAAC
>Dysdercus cingulatus
------------AGAATTTAATTTTAAGTCTGACCTGCTCAATGATTT------------
ATTAAATTGCTATAGTATTTTAACTATACAAAGGTAGCATAATAATTTGTCTTTTAATTG
GAGGCTTGTATGAATGGTTGGACGAGAAGTAAACTTTCTTAATTTAAAT--TAAATTGAA
TTTAATATTTATGTAAAAAAGCTTAAATATAA-T-TGTGGGACGAGAAGACCCTTTAGAA
TTTTAATTGAATT-AGTA------------ATTTT--TTATTTT----AGAAA-------
----------TTGTTTGTAATAT--TTTATTAATTTAATTTTTGTTGGGGTGACAGTAAA
AAT-TTTTTAACTTTTATTATTAAA---TTAA----TCATAAATT-----AGTGT----A
-TTA---TTGATCCTTTA-----T----ATAAAGATTAT--AAGATTAAATTACCTTAGG
GATAAC
>Pyrrhocoris apterus
---------ATGATCAATTAATTTTAAGTCTGACCTGCTCAATGAATT----------TA
TTTAAATTGCTATAGTATTTTAACTATACAAAGGTAGCATAATAATTTGTCTTTTAATTG
AAGGCTAGTATGAATGGTTGGACGAAAAATAAACTTTCTTAGTTAAAAT-TATA-TTGAA
TTTGATTTTTATGTAAAAAAGCTTAAATTTTA-T-TGTGGGACGAGAAGACCCTATAGAA
ATTTAATTAATAT-TTAA------------ATCCT--TTACTAT----AGTTTA------
----------TTTTATAAT----ATATTAATATATTAATTTATGTTGGGGTGACAGTAGA
AAA-TTGATAACTTCTATTTATTAA---ATTT----TCATAAATT-----AGTGT----A
-TTA---TTGATCCTTTT-----T-----TATGGATTAA--AAGATTAATTTACCTTAGG
GATAAC
>Physopelta gutta
-----------TTGTTAATAATTTTAAGTCTAGCCTGCTCAATGAATA--------TTTA
TTTTAATAGCCATAGTATTTTAACTATGCAAAGGTAGCATAATCATTTGTCTTTTAATTG
AAGGCTTGTATGAAGGGTTTGACGAGGGGTAGACTTTCTTAATTTAAAT--TTAATTAAA
CTTAACTTCTAGGTAAAAAGGCTTAGATATAG-T-TTTGGGACGAGAAGACCCTATAGAA
TTTTATTATTATA--------TT--------------TTATTTT----TGTTAT------
----------TATATTAATAAGA--GTTATTTGTAATAATTTTGTTGGGGTGACAGTGGA
ATT-TTTTTAACTTCTATTATT-TA---TATT----TCATTAATT-----AGTGT----T
-TTA---TTGATCCTTTA-----T-----TATGGATTAT--AAGATTAAATTACCTTAGG
GATAAC
>Acanthosoma nigrodorsum
-----------TGTTTATTTATATTAAGTCTTGCCTGCTCAATGATATTT-----ATTAT
ATTAAATAGCCGCAGTATATTGACTGTGCAAAGGTAGCATAATCATTTGTCTTTTAATTG
GAGACTTGTATGAATGGTTTGATGAGATATTGACTTTCTTTATATAAAA--TTA-ATTAA
CTTAATATTTTAGTTAAAAAGCTAAAATACTT-T-TAAGGGACGAGAAGACCCTATAGAA
TTTTATACTTTTG---------------AATTTAAT-TTATATT----TGGATA------
-----------TTTTTGAA-TTT--TATCTTTAAAAGTATTTTGTTGGGGTGACAGGGAA
ATT-TTCTTAACTTTCTTTATT-TA---TACT----TTATTAATA-----TATAT----A
-TTT---ATGATCCTATT-----T-----TATGGATATTTAAAGATTAAATTACCTTAGG
GATAAC
>Sastragala scutellata
---------TTGATTAATTTATATTAGGTTTTACCTGCTCAATGATAATT-----AAATT
ATTAAATAGCCGCAGTATATTGACTGTGCAAAGGTAGCATAATCATTTGTCTTTTAATTG
AGGACTTGTATGAATGGTTTGATGAGATATTGACTTTCTTTATATAAAA--TTA-AAAAA
ATTAATTTTTTAGTTAAAAGGCTAAAATAATT-T-TAAGGGACGAGAAGACCCTATAGAA
TTTTATACTTTTT-AAAT-------------TTATA-ATGTTTC----TGGTTA------
-----------TTTTTAAG-TTT--TATTTTTATAAGTATTTTGTTGGGGTGACAGGGAA
ATT-TCTTAAACTTTCTTTATT-TA---TACA----TTATTAATG-----AATAA----A
-TTT---TTGATCCTATT-----T-----TAAGGATTTT--AAGATTAAATTACCTTAGG
GATAAC
>Elasmostethus interstinctus
-----------TGTAAATTTATATTAAGTCTTGCCTGCTCAATGATAA-------TATTT

ATTAAATAGCCGCAGTATTTTGACTGTGCAAAGGTAGCATAATCATTTGTCTTTTAATTG
TGGACTTGTATGAATGGTTTGATGAGATATTAGCTTTCTTTATATAAAA--TTG-ATTAA
TTTAATTTCTTAGTTAAAAAGCTGAGATTATC-T-TATAGGACGAGAAGACCCTATAGAA
TTTTATGTATATA---------------TTTATAAA-TTATATT----TGGATA------
-----------TTTTTTAT-TTT--TATGGATTTATATATTTTGTTGGGGTGACAGGGAA
ATT-TATACAACTTTCTTTATT-TA---TATT----TTATTAATT-----TATAT----A
-TTT---ATGATCCTTTT-----T-----TAAGGATACT--AAGATTAAATTACCTTAGG
GATAAC
>Lindbergicoris hochii
----------TTGTATATTTATATTAAGTTTTACCTGCTCAATGATAT-------TAAAC
ATTAAATAGCCGCAGTATTTTGACTGTGCAAAGGTAGCATAATCATTTGTCTTTTAATTG
TGGACTTGTATGAATGGTTTGATGAAATATTTACTTTCTTTATCTAAAA--TCA-TTTAA
TTTAATCTTTTAGTTAAAAAGCTAAAATGACT-T-TATAGGACGAGAAGACCCTATAGAA
TTTTATAAATCTT---------------TATATAAT-TTATTTA----TAGTTA------
----------TTTTTAATA-AAA--TATATTTATATTTATTTTGTTGGGGTGACAGGGAA
ATT-TAATTAACTTTCTTTATT-AA---AATG----TTATTGATT-----TATAT----A
-TTT---ATAATCCTATT-----T-----TAAGGATATT--AAGATTAAATTACCTTAGG
GATAAC
>Elasmucha grisea
-----------TGTGTATTTATATTAAGTCATACCTGCTCAATGATATT------TAATT
ATTAAATGGCCGCAGTATTTTGACTGTGCAAAGGTAGCATAATCATTTGTCTTTTAATTG
TGGACTTGTATGAATGGTTTGATGAAATATTGACTTTCTTTATATAAAA--AAT-ATTAA
TTTAATATTTTAGTTAAAAAGCTAAAATTGAT-T-TATAGGACGAGAAGACCCTATAGAA
TTTTATACTTTTA-TTAA------------AACAAA-TAATAAT----TGGT--------
----------TTATTTATA-TTT--TGTTTTATGTAGTATTTTGTTGGGGTGACAGGAAA
ATT-TTTATAACTTTTCTTATT-TA---TTAT----TTATTTATT-----TGTAT----T
-TTA---ATGATCCTATT-----T-----TAAGGATATT--AAGATTAAATTACCTTAGG
GATAAC
>Elasmucha laeviventris
-----------TGTATATTTATATTAAGTCATACCTGCTCAATGATAAT------TAATT
ATTAAATGGCCGCAGTATTTTGACTGTGCAAAGGTAGCATAATCATTTGTCTTTTAATTG
TGGACTTGTATGAATGGTTTGATGAAATATTGACTTTCTTTATATAAAA--AAT-GTTAA
CTTAATATTTTAGTTAAAAAGCTAAAATTATT-T-TATAGGACGAGAAGACCCTATAGAA
TTTTATATTTTAT-ATAT------------AGTAAT-TAATATT----TGGTTG------
----------ATTATTTAT-TTT--ATTATAATAAAATATTTTGTTGGGGTGACAGGAAA
ATA-TCTAAAACTTTTTTTATT-TA---TTTA----TTATTTATT-----TATAT----T
-TTT---ATGATCCTATT-----T-----TAAGGATATT--AAGATTAAATTACCTTAGG
GATAAC
>Eusthenes cupreus
----------TGTTAAGTTTATATCAGGTCTGGCCTGCCCACTGATAA--------TTAT
ATTGAAGGGCCGCGGTATTTTGACCGTGCGAAGGTAGCATAATCATTTGTCTTTTAATTG
TAGGCTTGTATGAATGGTTTGATGAAATATAAACTTTCTTTTATTTATA--ATA-TTTAA
TTTAATTTTTTAGTTAAAAAGCTAAAATTGTC-T-TGTTGGACGAGAAGACCCTATAGAA
TTTTACTTGATCA-TTTA------------TATTTA-CTTTTTT----TGGTTA------
----------TTAATTTAA-GTG--TATATTATGTTAAGTTGTGTTGGGGCGACAGGGAA
ATT-TATTTAACTTTCTTTATT-TA---TATC----TTATTTATA-----TATAT----A
-TTA---ATGATCCTAAA-----T-----TTTGGATATT--AAGATTAAATTACCTTAGG
GATAAC
>Adrisa birmana
--------TTGTTAAAGTTTATATCAAGTCTGGCCTGCTCAATGATTTATATTT-TGGTG
ATTTAATAGCCGCAGTATTTTGACTGTGCAAAGGTAGCATAATCATTTGTCTTTTAATTG
TAGGCTTGTATGAATGGTTTGATGAGATACTAACTTTCTTTATATTAAT-ATTT-AAGAA
TTTAATATCTGAGTCAAAAAGCTTAGATTTAC-T-CGTAGGACGAGAAGACCCTATAGAA
TTTTATTATATTT-AATT------------TATTGT-TGGTTTT----TGGTTA------

----------TTATTAGTCTGAT--AATATTTTGTGTAATTGTGTTGGGGTGACAGGGAA
ATT-GTTTTAACTTTCTTTATT-TA---TTTA----TTACAGAAT-----TGTAT----A
-TTT---GAGATCCTATA-----A-----TTTGGATAGT--AAGATTAAATTACCTTAGG
GATAAC
>Adrisa magna
--------TTGGTAAAGTTTATATCAAGTCTGACCTGCTCAATGATTTATATTT-AAATG
ATTAAATAGCCGCAGTATTTTGACTGTGCAAAGGTAGCATAATCATTTGTCTTTTAATTG
TAGGCTTGTATGAATGGTTTGATGAAATATCAGCTTTCTTTATACTATT-ATTA-GAGAA
TTTAATATTTGAGTTAAAAAGCTTAAATGGTT-T-CGGAGGACGAGAAGACCCTATAGAA
TTTTATTATATTT-AATG------------TGTATT-TTACTTT----TGGTTT------
----------TTATTGATTAATT--TACATCTTTTGTAATTTTGTTGGGGTGACAGGGAA
ATT-TATTTAACTTTCTTTATA-TA---TTAT----TTACAGATT-----TGTAT----A
-TTT---ATGATCCTTTA-----T-----TTTGGATATT--AAGATTAAATTACCTTAGG
GATAAC
>Adrisa romani
--------TCGTTGAAGTTTATGTCAAGTCGGGCCTGCTCAATGATTTATATTG-TAATA
ATTTAATAGCCGCAGTATTTTGACTGTGCAAAGGTAGCATAATCATTTGTCTTTTAATTG
TAGGCTTGTATGAATGGTTTGATGAGATATCATCTTTCTTTATATTATC-ATGA-AAGAA
TTTAATTTTTAAGTTAAAAAGCTTAAATACTT-T-CGTAGGACGAGAAGACCCTATAGAA
TTTTATCATATTT-AATT------------CGTTCT-TATTTTT----TGGTTG------
----------TTATTAAATAAAT--TACGGGGTATATGATTATGTTGGGGTGACAGGGAA
ATT-AATTTAACTTTCTTTATT-TT---ATTA----TTACTGATT-----TGTAT----T
-TTT---ATGATCCTATT-----T-----TTTGGATAGT--AAGATTAAATTACCTTAGG
GATAAC
>Aethus pseudindicus
----------GGTGGGGATTAAATCATGTCGGGCCTGCTCATGATTTT---------AAT
ATTTAATAGCCGCAGTATCTTGACTGTGCGAAGGTAGCATAATCATTTGTCTTTTAATTG
TAGGCTTGTATGAAGGGTTTGATGAAATTCTAACTTTCTTTATTTTATA--TTT-AATAA
TTTAATGTTTGGGTCAAAAAGCTTAGATGATT-C-CATAGGACGAGAAGACCCTATAGAG
TTTTATTATGGGA-TTTG------------TGATTT-GTATTAT----GGTAAA------
----------TTTGTTTCA-TTT--TATAGATTCTATAATTGTGTTGGGGCGACAGGGAA
ATT-TTTTTAACTTTCTTTGAT-AA--TTTAT----TTATTAATG-----AGTAT----G
-TTT---ATGATCCCATA-----T-----TAGGGATAGT--AAGATTAAATTACCTTAGG
GATAAC
>Fromundus difficilis
----------TTGTATATTTTTATCAGGTCGGACCTGCTCAATGAT---------ATTAT
ATTAAATAGCCGCAGTATCTTGACTGTGCGAAGGTAGCATAATCATTTGTCTTTTAATTG
TAGGCTTGTATGAAGGGTTTGATGAAATTCTAGCTTTCTTTATTTTATA--TTT-AATAA
TTTAATGTTTAAGTCAAAAGGCTTAGATGATT-C-CATAGGACGAGAAGACCCTATAGAG
TTTTATTGTTATT-TACA------------TATT---TTGTTAT----AGGTAA------
----------TTGATTGCG-TTT--TATGTAAATGATAATTGTGTTGGGGTGACAGGGAA
ATT-TTATTAACTTTCTTTTTT-TA--TTTAT----TTATTGATGATTATAATAA----G
-ATA---ATGATCCTTTA-----T-----TATGGATTTT--AAGATCAAATTACCTTAGG
GATAAC
>Byrsinus pseudosyriacus
-----------TGTGAAATTTTATTAAGTCAGGCCTGCTCAATGATTA-------TATTG
ATTAAATAGCCGCAGTATTTTGACTGTGCGAAGGTAGCATAATCATTTGTCTTTTAATTG
TAGGCTAGAATGAATGGTTTGATGAAATCTTGACTTTCTTTATTTTAAT--ATT-AATAA
TTTAATGTTTGAGTCAAAAGGCTTAAATGATT-T-TGGAGGACGAGAAGACCCTATAGAA
TTTTATTGTAATT-TAGT------------AGTG---TTATTTT----AGGGAA------
----------ATGGTTAATGTCA--TTTATAATTTACAATTGTGTTGGGGTGACAGGGAA
ATT-AGTTTAACTTTCTCTATA-AGTATTAAT----ATGTTAATT-----GGCAT----A
TATT---TTGATCCTTTA-----T-----TAAAGATTAT--AAGATTAAATTACCTTAGG
GATAAC

>Byrsinus varians
----------TTGTAGAATTTTATTAAGTCGGGCCTGCTCAATGATTT--------ATTT
ATTAAATAGCCGCAGTATTTTGACTGTGCGAAGGTAGCATAATCATTTGTCTTTTAATTG
AAGGCTAGTATGAATGGTTTGATGAAATTTAGACTTTCTTTATTTTAAT--ATT-AATAA
TTTGATGTTTAAGTTAAAAAGCTAAGATTATT-T-CAGAGGACGAGAAGACCCTATAGAA
TTTTATTATTATT-TGAT------------AAAGAT-TTGTTTT----TGGGAG------
----------TTAATTTAA-TTT--TTTATTGATAATAATTTTGTTGGGGTGACGGGGAA
ATT-GTTTTAACTTTCTTTATA-AA---TGTATAATATGTTAATT---AGCATAT----A
-TAA---ATGATCCTTTA-----T-----TATAGATTAT--AAGATTAAATTACCTTAGG
GATAAC
>Fromundus pygmaeus
----------TTGTATAATTTTTTTAGGTCGGGCCTGCTCAGTGATTA--------TATA
ATTAAATAGCCGCAGTATTTTGACTGTGCGAAGGTAGCATAATCATTTGTCTTTTAATTG
TAGGCTAGTATGAAGGGTTTGATGAGGTGCTAACTATCTTAGTTTTATT--TTT-GGGAA
TTTAATGTTTAAGTTAAAAGGCTTAAATTTTT-T-CATAGGACGAGAAGACCCTATAGAG
TTTTACTTCTAGT-TTTG------------GATAAT-TTATCTT----TGGTAT------
----------TTAATTATA-TTT--TATTTGAGTAGGAGTTTTGTTGGGGTGACAGGGAA
ATT-TTACTAACTTTCTTTATT-TA--TATAT----TTAATTATG-----ATTAT----T
-TTA---AAGATCCTTTAATATTT-----TATGGATAGT--AAGATTAAATTACCTTAGG
GATAAC
>Geotomus convexus
----------TTGTATAATTTTATTAGGTCGGGCCTGCTCAGTGATTA--------TTTA
ATTAAATAGCCGCGGTATTTTGACTGTGCGAAGGTAGCATAATCATTTGTCTTTTAATTG
TAGGCTTGTATGAATGGTTTGATGAAGTACAAACTTTCTTTATTTCAAT--ATT-AAGAA
TTTAATGTTTAAGTCAAAAAGCTTAAATTTTT-C-CATAGGACGAGAAGACCCTATAGAA
TTTTACTCTTGTT-AAATG-----------AATAAT-TTATTTT----TGGTAT------
----------TTTATATAA-TTT--GTTTGTTTAAAGAGTTTTGTTGGGGTGACAGGGAA
ATT-TCAATAACTTTCTTTATA-AT---GTTT----TTATTTATG-----ATTAT----A
-TTA---ATGATCCTTTA-----T-----TAAGGATAAT--AAGATTAAATTACCTTAGG
GATAAC
>Microporus nigrita
----------TTGTATAAGTTTATTAAGTCAGGCCTGCTCAATGATTAT------AATTA
ATTAAATAGCCGCAGTATTTTGACTGTGCGAAGGTAGCATAATCATTTGTCTTTTAATTG
TGGGCTTGTATGAATGGTTTGATGAGATATTAACTTTCTTTATTCAATA--TTT-ACGAA
ATTAATACTTGAGTTAAAAAGCTTAAATGTTT-T-CGTGGGACGAGAAGACCCTATAGAG
TTTTATAATTATA-ATTA------------TTAAAT-TATTCAT----TGGTAA------
----------GTTTCATTA-ATT--CAATAGTTTAATTATTTTGTTGGGGTGACGGGAAA
ATT-TTATTAACTTTTCTTTAT-TT---TATA----TTAATAATT-----ATTAT----G
-TTTAGAATGATCCTTTA-----T-----TAAGGATAAT--AAGATTAAATTACCTTAGG
GATAAC
>Pangaeus bilineatus
----------TTGTGTAATTTTATTAGGTCGGGCCTGCTCAATGATTAAAGTTAAGGTAA
ATTAAATAGCCGCAGTATTTTGACTGTGCGAAGGTAGCATAATCATTTGTCTTTTAATTG
TAGGCTTGTATGAATGGTTTGATGAAATACTATCTTTCTTTACATTATA--ATA-TATAA
TTTAATTTTTTAGTTAAAAAGCTAAAATTTTT-T-CATAGGACGAGAAGACCCTATAGAG
TTTTATTGTATGA-TATA------------TTTTATTATTCTTT----TGGATA------
------TA--TTATTAGTTTAAT--AATTTATTTTACAATTGTGTTGGGGTGACAGGGAA
ATT-TTATTAACTTTCTTTATA-TA--TTTTA----TTATTAATT-----AGTAT----T
-TAA---ATGATCCTTTA-----T-----TATGGATAGT--AAGATTAAATTACCTTAGG
GATAAC
>Pangaeus rugiceps
----------TTGTGGAATTTTATTAGGTCGGGCCTGCTCAATGATTAAATTTA-ATATA
ATTAAATAGCCGCAGTATTTTGACTGTGCGAAGGTAGCATAATCATTTGTCTTTTAATTG
TAGGCTTGTATGAATGGTTTGATGAAATACTATCTTTCTTTACATCATA--ATT-TATAA

TTTAATTTTTTAGTTAAAAAGCTAAAATTTTT-T-CATAGGACGAGAAGACCCTATAGAG
TTTTATTGTATGA-TATA------------TTTTATTATTCTTT----TGGATA------
------TG--TTATTAGTTTAAT--AATTTGTTTTACAATTGTGTTGGGGTGACAGGGAA
ATT-TTATTAACTTTCTTTATA-TA--TTTTA----TTATTGATT-----AGTAT----T
-TTA---ATGATCCTTTA-----T-----TATGGATAGT--AAGATTAAATTACCTTAGG
GATAAC
>Rhytidoporus indentatus
----------TGTTTGTATAATATCAGGTCAGACCTGCTCAATGATTAGAAG---TGTTA
ATTAAATAGCCGCAGTATTTTGACTGTGCGAAGGTAGCATAATCATTTGTCTTTTAATTG
TAGGCTTGTATGAATGGTTAGATGAAACATTAGCTTTCTTTACATCAAT--TTT-TATAA
TTTAATGTTTGAGTCAAAAAGCTTAAATTCTT-T-CATAGGACGAGAAGACCCTATAGAA
TTTTATTATCATA-TATT------------TTATT--ATATTAT----GGGTAT------
----------TTATTATTA-TTT--TGTAATAGTGGTAATTGTGTTGGGGTGACAGGGAA
AAT-GTATTAACTTTCTTTATT-TA-TTGTGT----TTATTGATT-----TATAT----A
-TTA---ATGATCCTTTA-----T-----TAAGGATATA--AAGATTAAATTACCTTAGG
GATAAC
>Macroscytus annulipoides
----------TTGTTAAATTATATTAAGTCAGGCCTGCTCAGTGATTAT------TAATA
ATTAAATAGCCGCAGTAATTTGACTGTGCGAAGGTAGCATAATCATTTGTCTTTTAATTG
GAGGCTTGTATGAATGGTTTGATGAAACTCTAGCTTTCTTTATTGTAAA---TT-TAGAA
TTTAATATGTGAGTTAAAAAGCTTAGATATTT-C-CATAGGACGAGAAGACCCTATAGAA
TTTTACTTATATT-TATA------------AATTTA-TTAGTAT---AGGGTGA------
---------TTTTATTACA-GAT--AAGTTTTATATAAATTGTGTTGGGGTGACAAAGAA
ATT-AATTTAACTTTCTTTTAT-----GTTTA----TTAATTATG-----TTTAT----A
-TTT---ATGATCCTTTT-----T-----TATGGATAGT--AAGATTAAATTACCTTAGG
GATAAC
>Macroscytus badius
--------TTGTTATAAATTATATCAAGTCAGGCCTGCTCAGTGATTAT------ATAGA
ATTAAATAGCCGCAGTAATTTGACTGTGCGAAGGTAGCATAATCATTTGTCTTTTAATTG
AAGGCTTGTATGAATGGTTTGATGAAACTCTGACTTTCTTCACTGTAAA--TGT-TAAAA
TTTAATATTTGAGTTAAAAAGCTTAGATATTT-C-CATAGGACGAGAAGACCCTATAGAA
TTTTACTGTATGT-TTAA------------TATTAT-TTATTTT----AGGTAT------
----------TTTATTTTTGAGA--ATTTTAAATTACAGTTATGTTGGGGTGACAAAGAA
ATT-AACTTAACTTTCTTTATT-TT--TGATT----TTAATGATT-----TTTAT----A
-TTT---ATGATCCTTTA-----T-----TATGGATAGT--AAGATTAAATTACCTTAGG
GATAAC
>Macroscytus brunneus
--------TTGTTTTAAATTATATTAAGTCAGGCCTGCTCAGTGATTAT------ATAGA
ATTAAATAGCCGCAGTAATTTGACTGTGCGAAGGTAGCATAATCATTTGTCTTTTAATTG
AAGGCTTGTATGAATGGTTTGATGAAACTCTGACTTTCTTCACTGCAAA--GAT-TAGAA
TTTAATATTTGAGTTAAAAAGCTTAGATATTT-C-CATAGGACGAGAAGACCCTATAGAA
TTTTACTATATAT-TTAA------------TATTAT-TTATTTT----AGGTAT------
----------TTTATTTTTAAGA--ATTTTAGATTATAGTTGTGTTGGGGTGACAAAGAA
ATT-AATTTAACTTTCTTTATTATT--TGATT----TTAATGATT-----TTTAT----A
-TTA---ATGATCCTTTA-----T-----TATGGATAGT--AAGATTAAATTACCTTAGG
GATAAC
>Macroscytus fraterculus
---------TTGTTAATATTATATTAGGTCAAGCCTGCTCAGTGATTA-------TTATT
ATTAAATAGCCGCAGTAATTTGACTGTGCGAAGGTAGCATAATCATTTGTCTTTTAATTG
GAGGCTTGTATGAATGGTTTGATGAAACTTTAACTTTCTTTGCTATAAA--TAT-TAGAA
TTTAATGTTTGAGTTAAAAAGCTTAGATATTT-C-CATAGGACGAGAAGACCCTATAGAG
TTTTACTATAATT-ATGA------------TTTAT--TTAAATT----AGGTAT------
----------GTATTTTTAAATA--TTTACTTTTTATATTTATGTTGGGGTGACAGAGAA
ATT-AATATAACTTTCTTTATT-TA--TATTG----TTATTGATG-----TTTAT----A

-TTA---TTGATCCTTTA-----T-----TATGGATAAT--AAGATTAAATTACCTTAGG
GATAAC
>Macroscytus japonensis
---------TTGTTAATATTATATTAGGTCAAGCCTGCTCAGTGATTA-------TTATT
ATTAAATAGCCGCAGTAATTTGACTGTGCGAAGGTAGCATAATCATTTGTCTTTTAATTG
GAGGCTTGTATGAATGGTTTGATGAAACTTTAACTTTCTTTGCTATAAA--TAT-TAGAA
TTTAATGTTTGAGTTAAAAAGCTTAGATATTT-C-CATATGACGAGAAGACCCTATAGAG
TTTTACTATAATT-ATGA------------TTTAT--TTAAATT----AGGTAT------
----------GTATTTTTAAATA--TTTACTTTTTATATTTATGTTGGGGTGACAGAGAA
ATT-AATATAACTTTCTCTATT-TA--TATTG----TTATTGATG-----TTTAT----A
-TTA---TTGATCCTTTA-----T-----TATGGATAAT--AAGATTAAATTACCTTATG
GATAAC
>Macroscytus minimus
----------TTGAATAAGAATATTAGGTCAGGCCTGCTCAATGATTAAT-----TATTG
ATTAAATAGCCGCAGTAATTTGACTGTGCGAAGGTAGCATAATCATTTGTCTTTTAATTG
GAGGCTTGTATGAATGGTTTGATGAAGTACCTACTTTCTTTACTATAAA--GGT-TAAAA
TTTAATATTTGAGTTAAAAAGCTTAGATATTT-C-CATAGGACGAGAAGACCCTATAGAG
TTTTACTGTATTG-GTGA------------ATTACT-TTATA------AGGTAA------
---------TGTATTTTAA-GGA--GTTTACACTATCAGTTATGTTGGGGTGACAAAGGA
ATT-TTAATAACTTCCTTTATTAGA---TGTT----TTATTGATA-----TATAT----G
-TAT---ATGATCCTTTA-----T-----TATGGATATT--AAGATTAAATTACCTTAGG
GATAAC
>Adomerus biguttatus
------------TGTTATTTATTTTAAGTCAAACCTGCTCAATGATAT--------TATT
ATTAAATAGCCGCAGTATTTTGACTGTGCAAAGGTAGCATAATCATTTGTCTATTAATTG
TAGGCTGGTATGAATGGTTGGATGAAATATTGACTTTCTTTATTTTTATAATTA-ATGAA
TTTAATATTTAAGTTAAAAAGCTTAAATAATT-T-TGTAGGACGAGAAGACCCTGTAGAA
TTTTATTTAAATT-TTAA------------TTAATT-GTAATTT----T-----------
----------ATTAATTAT-ATT--TATTAATTCTTAAATTTTGTTGGGGTGACAGGGAA
AAT-CTAATAACTTTCTTTATTATT---ACTT----TTATTAATT-----AATAT----A
-TTA---AAAATCCTTTA-----T-----TAAAGA-AGT--AAGATTAAATTACCTTAGG
GATAAC
>Adomerus variegatus
----------TGTTATATTTATTTTAGGTCAAGCCTGCTCAATGATTA----------TT
ATTAAATAGCCGCAGTATTTTGACTGTGCAAAGGTAGCATAATCATTTGTCTATTAATTG
TAGGCTGGTATGAATGGTTAGATGAAATATTGACTTTCTTTGCTCTATA--TAA-TTGAA
TTTAATATTTAAGTTAAAAAGCTTAAATGGTT-T-TGTAGGACGAGAAGACCCTATAGAA
TTTTATTTAATTA-TTAA------------TTTGTT-ATGATTT------AGTT------
-----------ATTATTGT-ATA--AATTGGTGATTAAATTTTGTTGGGGTGACAGGGAA
AAT-TTAATAACTTTCTTTATT-TA---TTAT----TTATTAATT-----GGTAT----A
-TTA---AAGATCCTTTA-----TT----TAAGGATTAT--AAGATTAAATTACCTTAGG
GATAAC
>Sehirus luctuosus
-------TTGTTATATTTTTATTTTAAGTCAAGCCTGCTCAATGATTA--------ATTA
ATTAAATAGCCGCAGTATTTTGACTGTGCAAAGGTAGCATAATCATTTGTCTATTAATTG
TAGGCTGGTATGAATGGCTAGATGAAATATTAACTTTCTTTATTTTATATTTAT-TTGAA
TTTAATTTTTAGGTTAAAAAGCTTAAATGTTTAT-TGTAGGACGAGAAGACCCTATAGAA
TTTTATTTAATTA-TTGG------------TTTGAT-ATAAATT------ATTT------
----------GATTATTAT-TTA--AATTAATAATTAAATTTTGTTGGGGTGACAGGGAA
AAT-ATAATAACTTTCTTTATT-TA---TTAT----TTATAAATT-----AATAT----A
-TTA---AAAATCCTTTA-----T-----ATAAGGAAAT--AAGATTAAATTACCTTAGG
GATAAC
>Canthophorus niveimarginatus
----------TGAGATATTGATTATAGGTCAGGCCTGCTCAGTGATTATT------AAAA

ATTAAATAGCCGCAGTATTTTGACTGTGCAAAGGTAGCATAATCATTTGTCTATTAATTG
TAGGCTAGTATGAATGGTTTAATGAGATATTAACTTTCTTTACTTTACATTTAT-TTGAA
TTTAATATTTAAGTTAAAAAGCTTAGATGTTT-T-TGTGGGACGAGAAGACCCTATAGAA
TTTTATTTAATTA-TTGA------------TTTATT-TTTAATT------TATT------
----------AATCATTAA-AAA--ATTATGTAATTAAATTTTGTTGGGGTGATAGGGAA
AAT-TTCATAACTTTCTTTATA-TA---TTTG----TTATAAATT-----AATAT----A
-TTA---AATATCCTTTA-----T-----TAAGGA-AAT--AAGATTAAATTACCTTAGG
GATAAC
>Legnotus limbosus
--------TTGTTTATTTTTATTTTAAGTCAATCCTGCTCAATGATCATT------TATG
ATTAAATAGCCGCAGTATTTTGACTGTGCAAAGGTAGCATAATCATTTGTCTGTTAATTA
TAGGCTGGTATGAACGGATAAATGAAATATTAACTTTCTTTATTTTATA--TTT-ATGAA
TTTAATTTTTAGGTTAAAAAGCTTAAATGTTT-T-TGTAGGACGAGAAGACCCTATAGAA
TTTTATTTATTTG-TTGA------------TTAGTT-TAATTTT----------------
--T------TTATTATTAT-TAT--TTTTAATAGATGAATTTTGTTGGGGTGACAGGGAA
AAT-TTTATAACTTTCTTTATT-AA---TTGT----TTATTGATT-----TATAT----G
-TTA---AGGATCCTTTA-----T-----TAGGGA-AGT--AAGATTAAATTACCTTAGG
GATAAC
>Tritomegas bicolor
---------TTGAATTTTTGATTTTAAGTCAAGCCTGCTCAATGAT----------TATT
ATTAAATAGCCGCAGTATTTTGACTGTGCAAAGGTAGCATAATCATTTGTCTATTAATTG
TAGGCTGGTATGAATGGTTAGATGAAATACTAGCTTTCTTTATTTTATGTTTTG-ATGAA
TTTAATATTTGAGTTAAAAAGCTTAAATGTTT-T-TGTAGGACGAGAAGACCCTATAGAA
TTTTACTTAATTG-ATTA------------TTTAAT-TAGATTT----T-----------
----------TTATATTTA-TTA--AATTTTTAGTTGAGTTTTGTTGGGGTGACAGGGAA
AAT-TCAGTAACTTTCTTTATT-AA---ACAT----TTATTAATT-----GGTAT----A
-TTA---AATATCCCTTT-----T-----TAGGGA-AAT--ATGATTAAATTACCTTAGG
GATAAC
>Tritomegas sexmaculatus
--------TTGTTATTTTTATTTTAAGTCAAGCCTGCTCAATGATT--------CTATT
ATTAAATAGCCGCAGTATTTTGACTGTGCAAAGGTAGCATAATCATTTGTCTATTAATTG
TAGGCTGGTATGAATGGTTAGATGAAATATTGACTTTCTTTATTTTATT-ATTT-ATGAA
TTTAATGTTTGAGTTAAAAAGCTTAAATATTT-A-TGTAGGACGAGAAGACCCTATAGAA
TTTTACTTAATTTAATTA------------TTTGGT-TAAATTT----T-----------
----------TTATATTTA-TTA--AATTTTTATTTAAGTTTTGTTGGGGTGACAGGGAA
AAT-TTAATAACTTTCTTTATT-AA---GTTT----TTATTGATT-----TATAT----A
-TTA---AATATCCCTTT-----T-----TAGGGA-AAT--ATGATTAAATTACCTTAGG
GATAAC
>Crocistethus waltlianus
------TTATAGTTAGATTTATTTTAAGTCAGGCCTGCTCAATGATTAAT-----ATTGA
ATTAAATAGCCGCAGTATTTTGACTGTGCAAAGGTAGCATAATCATTTGTCTATTAATTG
TAGACTGGTATGAATGGTTAGATGAGATATTAACTTTCTTTATTTTATATTTATGTTGAA
CTTAATTTTTGAGTTAAAAAGCTTAAATGTTT-T-AATAGGACGAGAAGACCCTATAGAA
TTTTATTTAATTA-GTTA------------ATTAAG-TTTAATA----------------
----------TTTAATTAA-ATA--TTTTGCTAATTAAATTTTATTGGGGTGATAGGGAA
AAT-TAAGTAACTTTCTTTATT-AA---TTAT----TTATTAATT-----AGTAT----A
-TTA---AAGATCCCTTG-----T-----TTGGGA-ATT--AAGATTAAATTACCTTAGG
GATAAC
>Adomerus rotundus
-------------TGAATTTATTTTAAGTCAAACCTGCTCAATGATTG-------ATAAT
ATTAAATAGCCGCAGTATTTTAACTGTGCAAAGGTAGCATAATCACTTGTCTATTAATTA
TAGGCTGGTATGAATGGTTTAATGAAATATTAACTTTCTTTATTTAAC---TTT-ATGAA
TTTAATATTTGAGTTAAAAAGCTTAAATATTTAT-TGTAGGACGAGAAGACCCTATAGAA
TTTTATTTATTAA-CCAT------------TTTGTT-TATATTA----------------

```
----------TTTTATTAA-ATT--TATTGGTTACTAAATTTTATTGGGGTGATAGGGAA
ATT-TACTTAACTTTCTTTATT-TA---TATA----TTATTAAAT-----TATAA----A
-TTA---AAAATCCTTTT-----T-----TAAGGA-AAT--ATGATTAAATTACCTTAGG
GATAAC
>Adomerus triguttulus
----------TGTAAAGTTTATTTTAAGTCAGACCTGCTCAATGATCAA------ATAGT
ATTAAATAGCCGCAGTATTTTAACTGTGCAAAGGTAGCATAATCATTTGTCTATTAATTG
TAGGCTGGTATGAATGGTTTAATGAAATATTGACTTTCTTTATTTAAT---TTA-GTGAA
TTTAATATTTAAGTTAAAAAGCTTAATTGTTT-T-TGTAGGACGAGAAGACCCTATAGAA
TTTTATTTAGTAA-CCAT------------TTTAGT-TTAATTT----------------
---------GTTTAATTAA-ATT--TATTGATAACTAAATTTTGTTGGGGTGACAGGGAA
ATT-TAATTAACTTTCTTTAAT-TA---AATC----TTATTAGTT-----AATAT----T
-TTA---AAAATCCTTTA-----T-----TTAAGGAATT--ATGATTAAATTACCTTAGG
GATAAC
>Ochetostethus brachyscytus
--------TTGTTAATATTTATTTTAAGTTAAGTCTGCTCAATGATTGAT-----ATTAT
ATTAAATAGCCGCAGTATTTTGACTGTGCAAAGGTAGCATAATCATTTGTCTTTTAATTG
TGGGCTAGTATGAATGGCTAGATGAAATATTGACTTTCTTTATTTTATATTTGT-ATGAA
TTTAATTTTTATGTTAAAAAGCTTAAATGTTT-T-TGCGGGACGAGAAGACCCTATAGAA
TTTAATTTATATA-GTTT------------ATTTCA-TAATTTT----TGAGTT------
----------TATTTTAAT-GTT--GATTGTTATATAAATTTTGTTGGGGTGACAGGGAA
AAT-TAATTAACTTTCTTTACT-TA---TTGT----TTATTGATT-----TATAT----A
-TTA---ATGATCCTTTT-----T-----TAAGGATAAT--AAGATTAAATTACCTTAGG
GATAAC
>Ochetostethus heissi
--------TTGTTAATTTTTATTTTAAGTCAAGCCTGCTCAATGATTAT------TGAAA
TTTTAATAGCCGCAGTATTTTGACTGTGCAAAGGTAGCATAATCATTTGTCTTTTAATTG
TAGGCTGGTATGAATGGTTAGATGAAATATTAGCTTTCTTTATTTTATAATTGA-ATGAA
TTTAATGTTTGGGTTAAAAAGCTTAAATGTTT-T-TGTAAGACGAGAAGACCCTATAGAA
TTTTATTTATCTA-TTTT------------GATTAT-TTTATTA----TTATTG------
-----TTG--GTTATTATA-ATT--AACAAAATGGAAAATTTTGTTGGGGTGACCGGGAA
AAT-TTATTAACTTTCTTTATT-AA---ATTA----TTATTGATG-----TATAT----A
-TAT---AAGATCCTTTA-----T-----TAAGGATAAT--AAGTTTAAATTCCCTTAGG
GATAAC
>Ochetostethus opacus
--------TTGTTAATTTTTATTTTAAGTCAAGCCTGCTCAATGATTAT------TGAAA
TTTTAATAGCCGCAGTATTTTGACTGTGCAAAGGTAGCATAATCACTTGTCTTTTAATTG
TAGGCTGGTATGAACGGTTAGATGAAATATTAGCTTTCTTTATTTTATAATTGA-ATGAA
TTTAATATTTGGGTTAAAAAGCTTAAATGTTT-T-TGTAAGACGAGAAGACCCTATAGAA
TTTTATTTTCTTA-TTTT------------GATTAT-TTTATTA----TTATTG------
-----TTG--TTTATTATA-ATT--AACAAAATGGAAAATTTTGTTGGGGTGACAGGGAA
AAT-TTATTAACTTTCTTTATA-AA---ATTA----TTATTGATG-----TATAT----A
-TGT---AAGATCCTTTA-----T-----TAAGGATAAT--AAGGTTAAATTACCTTAGG
GATAAC
>Ochetostethus nanus
--------TTGTTAATATTTATTTTAAGTCAAGCCTGCTCAATGATTATT-----TTTAG
ATTTAATAGCCGCAGTATTTTGACTGTGCGAAGGTAGCATAATCATTTGTCTTTTAATTG
TAGGCTGGTATGAATGGTTAGATGAAATATTGACTTTCTTTATTTTATAATTGA-ATGAA
CTTAATATTTAGGTTAAAAAGCTAAAATGTTT-T-TATAAGACGAGAAGACCCTATAGAA
TTTTATTTATTTG-ATTT------------GGTAAT-TTTATTA----TTATTG------
-----TTG--ATTATTTAT-AGT--TATTGATTGATTAATTTTGTTGGGGTGACAGGGAA
AAT-TAATTAACTTTCTTTATT-AA---AGTA----TTACTGATT-----TGTAT----A
-TAA---AAGATCCTTTA-----A-----TAAGGATAAT--AAGATTAAATTACCTTAGG
GATAAC
```

>Ochetostethomorpha secunda
--------TTGATAATATTTATTTTAAGTCTAACCTGCTCAATGACTTG------AAAAA
GTTTAATAGCCGCAGTATTCTGACTGTGCAAAGGTAGCATAATCATTTGTCTTTTAATTG
TAGGCTAGTATGAAGGGTTAAATGAGATATTAACTTTCTTTGTTATAAATATTT-ATGAA
TTTAATTTTTAAGTTAAAAAGCTTAAATGTTA-T-TGTGGGACGAGAAGACCCTATAGAA
TTTAATTTATTTA-ATTAATGTTGACAATTATTAAT-TATTTTT----GTAAAT------
----------GTTATTTAT-CAT--TATTAATAAATAAATTTTGTTGGGGTGACAGGGAA
TATATATTTAACTTTCTTTATT-TAAATCATG----TTATTAATC-----TATAT----A
-TTA---ATGATCCTTTA-----T-----TAAGGATAAT--AAGATTAAATTACCTTAGG
GATAAC
>Amaurocoris curtus
----------AGTTATATTTGTTTTAAGTCTGTCCTGCTCAATGATTT-------ATTTA
ATTAAATGGCCGCAGTATTTTGACTGTGCAAAGGTAGCATAATCATTTGTCTTTTAATTG
TAGGCTTGTATGAATGGTTTGATGAAATACCAGCTTTCTTTATATTATA---TA-GTGAA
TTTAATTTTTGAGTTAAAAAGCTTAAATTTAT-T-TATGGGACGAGAAGACCCTATAGAA
TTTTATTTATATT-TAAT------------TTATTT-AATTATA----TGGT--------
----------TATTTTAATTATT--AGTTTAAATATAAATTATGTTGGGGCGACAGGAAA
ATT-TAATTAACTTTTCTTATT-TA---TATT----TTATATATT-----TATAT----A
-TTA---AAGATCCTAGA-----A-----TTAGGATTAT--AAGATTAAATTACCTTAGG
GATAAC
>Galgupha australis
-------AGGGTTAATATTTATTTTAGGTCTGGCCTGCTCAATGATCTAT-----TATGG
ATTAAATGGCCGCAGTATTTTGACTGTGCGAAGGTAGCATAATCATTTGTCTTTTAATTG
AAGGCTTGTATGAATGGTTTAATGAGACACACACTTTCTTCATATCATATATTA-TTTAA
TTTAATGTTTGAGTTAAAAAGCTTAAATTTAT-T-TATAGGACGAGAAGACCCTATAGAA
TTTTGATTTATAT-ATTA------------TATATT-TAATGGTTTGGTTATTT------
----------CTCATTAATGTTT--ATTGTTATAGATAATTTTGTTGGGGCGACAGGGAA
ATT-TTAGTAACTTTCTTTATT-TA---TATA----TTATTTATT-----TATAT----A
-TTA---ATGATCCTATT-----T-----TA-GGATAAT--AAGATTAAATTACCTTAGG
GATAAC
>Galgupha difficilis
TGTCCTATTGATTATTATTTATTTTAGGTCTGGCCTGCTCAATGATTAGTGATT-ATTAA
ATTAAATGGCCGCAGTATTTTGACTGTGCGAAGGTAGCATAATCATTTGTCTTTTAATTG
AAGGCTTGTATGAATGGTTTGATGAGATACATACTTTCTTCATATCATATACTA-TTGAA
TTTAATATTTGAGTTAAAAAGCTTAAATGTTT-T-TGTAGGACGAGAAGACCCTATAGAA
TTTTATTATTTAT-TTAT-----------ATTTTAT-TTATTTT---GGTATAT------
----------TTATTTATCTGGG--ATATTTATAGATTATTTTGTTGGGGCGACAGGGAA
ATT-TTAATAACTTTCTTTATT-TA---TATT----TTATTTATT-----TATAT----T
-TTA---CTGATCCTTGA-----T-----TA-GGATAAC--AAGATTAAATTACCTTAGG
GATAAC
>Eurygaster maura
---------AAAGTTTATTTATTTCAAGTCGGGCCTGCTCAATGATTTA------TAATA
ATTAAATAGCCGCAGTATTTTGACTGTGCGAAGGTAGCATAATCATTTGTCTTTTAATTG
AAGGCTTGTATGAAGGGTTTGACAAAATGTGGGCTTTCTTTACATTAAA--TCG-TTGAA
TTTAATTTTTGAGTTAAAAAGCTTAAATGTTT-T-AATAGGACGAGAAGACCCTATAGAA
TTTTATTATTTGT-GGTT------------TATA---TTATAAT----TGGTTT------
----------ATTTATTTA-TTA--TATGTTATAAATAATTTTGTTGGGGTGATGGGGAA
ATT-TTCTTAACTTTCTTTATT-TA---TTTA----ATATAAATT-----AATAT----A
-TTA---ATGATCCTTTT-----------TATGGATAAT--AAGTTTAAATTACCTTAGG
GATAAC
>Odontoscelis fuliginosa
----------GGGATTATTTTTCTTAAGTCGGGCCTGCTCAATGATTT----------TT
ATTAAATAGCCGCAGTATTTTGACTGTGCGAAGGTAGCATAATCATTTGTCTTTTAATTG
AAGGCTTGTATGAAGGGTTTGACGAAATGCAATCTTTCTTTGTATCAAA---TA-TTGAA

```
TTTAATTTTTAGGTTAAAAAGCTTAAATGTTT-T-TATGGGACGAGAAGACCCTATAGAA
TTTTATTATTTTA-TGTA------------TTGGAT---AATTT----TGGTGT------
----------ATTTAATTA-TTT--ATTATGTAAAATAATTTTGTTGGGGTGACATGGAA
ATT-TCTTTAACTTTCTTTACT-TA---TTTT----ATATTGATT-----AATAT----T
-TTT---AAGATCCTAAG-----T-----TTATGGAAAT--AAGTTTAAATTACCTTAGG
GATAAC
>Poecilocoris nepalensis
------------TGTAATTGATTTCAAGTCGGACCTGCTCAATGATTACCTTTATAGGGT
ATTAAATAGCCGCAGTATTTTGACTGTGCGAAGGTAGCATAATCATTTGTCTTTTAATTG
TAGGCTTGTATGAATGGTTTAACAAAATGTAATCTTTCTTTATATTAAT--ATT-ATGAA
TTTAATTTTTGAGTTAAAAAGCTTAAATTTAT-T-TATAGGACGAGAAGACCCTGTAGAA
TTTTACTTAATAT-GTAT------------TATTTT---AATTT----TGGTTA------
----------TTTATTTTA-TTT--TATTTTATATTAAGTTTTATTGGGGCGATAGGGAA
ATT-TTATTAACTTTCTTTTTT-TA---TTTA----TTATTAATT-----TGTAT----A
-TTA---TTGATCCTTA------T-----TTTGGATAAA--AAGATTAAATTACCTCAGG
GATAAC
>Thyreocoris scarabaeoides
----------TTGTTTATTTATTTTAGGTCAGGCCTGCTCAATGATTAATT----TTTTA
ATTAAATGGCCGCAGTATCTTGACTGTGCAAAGGTAGCATAATCATTTGTCTTTTAATTG
TAGGCTTGTATGAATGGCTCGATGAGGTATAAACTTTCTTTATATTAAA---TG-TTGAA
TTTAATTTTTAAGTTAAAAGGCTTAAATGTTT-T-TAGAGGACGAGAAGACCCTATAGAG
TTTTACTCTACAA-TCAT------------TATT---CAATTTT----TGGATA------
----------ATTGTAGTT-GAA--TAATGGTTTAGGAGTTTTGTTGGGGCGACAGGGAA
ATT-TTATTAACTTTCTTTATG-TA---TTAT----TTATTAATT-----AGTAT----A
-TTA---AAGATCCTGGA-----T-----TA-GGATAAT--AAGATTAAATTACCTTAGG
GATAAC
>Chilocoris neozealandicus
---------TGATATTATTGATATTAAGTCTGACCTGCTCAATGATTG-------ATTTA
ATTAAATAGCCGCAGTATTTTGACTGTGCAAAGGTAGCATAATCATTTGTCCTTTAATTG
TGGGCTAGAATGAATGGTTTGATGAAATATAAACTTTCTTTATTATAATTATCA-TAGAA
TTTAATATTTTAGTTAAAAAGCTAAAATGGTT-T-TATGGGACGAGAAGACCCTATAGAA
TTTTATATGTTAT-AATA------------ATTAAT-TTGTTTA----AGTGAAA-----
----------ATATATGAA-ATT--AATTATTATGATTATTATGTTGGGGTGACAGGGAA
ATT-TAATAAACTTTCTTTATT-TA---ATTT----TTATTGATT-----AATAT----A
-TTA---AAGATCCTTTA-----C-----GTAGGATTAT--AAGATTAAATTACCTTAGG
GATAAC
>Dismegistus sanguineus
---------TGTGATATTTTATTTTAAGTCAAACCTGCTCAATGATTT----------AT
ATTAAATAGCCGCAGTATTTTGACTGTGCAAAGGTAGCATAATCATTTGTCTTTTAATTG
TAGGCTGGAATGAATGGTTAGATGAAATATTAACTTTCTTTATTTTATA-TTGA-AAGAA
TTTAATTTTTTAGTTAAAAAGCTAAAATATTT-C-TGTAAGACGAGAAGACCCTATAGAA
TTTTACTTAATAT-ATAT------------TAGT---TGCATCT----TAAATA------
----------TTACCATGT-GTT--AAACACATATTAAGTCTCGTTGGGGTGATGGGGAA
ATT-TTATTAACTTTCTTTATA-TA---TAAT----TTACTAATTT---GTATAT----A
-TTA---AAGATCCTTAT-----A-----TTAAGATAAT--AAGATTAAATTACCTTAGG
GATAAC
>Chilocoris confusus
----------TGAGTATTTAATTTTAAGTCTTGCCTGCTCAATGATAA--------AATA
ATTAAATAGCCGCAGTATTTTGACTGTGCAAAGGTAGCATAATCATTTGTCCTTTAATTG
GGGGCTAGTATGAATGGTTTGATGAAATATAAACTTTCTTTATTATAATGGGTA-TTGAA
TTTAATTTTTTAGTTAAAAGGCTGAAATAGTT-T-TATGGGACGAGAAGACCCTATAGAA
TTTTATTTATTTT-TATT------------TTTTAC-TTACTATT---GGGTTA------
----------GTAATTATT-TTA--TAATAAATAGTAAATTTTGTTGGGGTGACAGGGAA
ATT-TTAATAACTTTCTTTAAA-AT---TAGT----TTACTAATT-----AGTAT----A
```

-ATA---ATGATCTTTCA-----T-----TGGTGATTAA--AAGACTAAATTACCTTAGG
GATAAC
>Chilocorus capensis
----------TGATAAATTAATATTAAGTCTTACCTGCTCAATGATAT--------TAGT
ATTAAATAGCCGCAGTATTTTGACTGTGCAAAGGTAGCATAATCATTTGTCCTTTAATTG
TGGGCTAGAATGAATGGTTTGATGAAATATAATCTTTCTTTATTATAATAATTA-TTGAA
TTTAATATTTTAGTTAAAAAGCTGAAATATTT-T-TAAGGGACGAGAAGACCCTATAGAA
TTTTATATTTTAT-ATTC------------AAAATA---------TAAAAATGT------
----------TAAAATAAGAATC--TTGAATATAAAATATTATGTTGGGGTGACTGGGAA
ATA-TTTAAAACTTTCTTTATT-AA---TTAA----TTGTTAATT-----AGCAT----A
-TTT---ATGATCTTATA-----A-----TATAGATTTT--AAGAGAAAATTACCTTAGG
GATAAC
>Parachilocoris minutus
-----TGATAATATATTTAAATAATAAGTCTGACCTGCTCAATGATAT----------AT
ATTAAATAGCCGCAGTATTTTGACTGTGCAAAGGTAGCATAATCATTTGTCCTTTAATTG
TGGGCTTGTATGAATGGTTTGATGAAATATAATCTTTCTTTATTATAATAATTA-TTGAA
TTTAATTTTTTGGTTAAAAAGCTAAAATGTTT-T-TATGGGACGAGAAGACCCTATAGAA
TTTAATAATTTTT-ATTT------------ATTAAA-TTTAAAT----TATGTT------
----------AAAATTATA-ATT--TATAGATAAATTTATTATGTTGGGGTGACAGGGAA
ATT-AATTAAACTTTCTTTTAT-TA--ATAAA----TTATAAATT-----AGTATAT--A
TTTA---ATGATCTTATA-----T-----TATAGATTAA--AAGAATAAATTACCTTAGG
GATAAC
>Blaena setosa
-----------TTGCAATTTATATCACGTTTGGCCTGCTCAATGAATA---------ATT
TTTAAATAGCCGCAGTACTTTGACTGTGCGAAGGTAGCATAATCATTTGTCTTTTAATTG
AAGGCTTGTATGAATGGTTGGATGAAATATATGCTTTCTTTATTTTATA--TAT-TAGAA
TTTAATTTTTGAGTTAAAAAGCTTAAATTTGT-T-TAGAGGACGAGAAGACCCTATAGAG
TTTTATATATTTT-TTAT------------TAAGTA-CTATTTG----------------
----------TTATTAGTA-CAT--TATAATTTATTATATTTTGTTGGGGCAATGGGGAA
ATT-TTAATAATTTTTCTTATT-TA---TTTT----TTATTTATT-----TGTAA----T
-TTA---AGGATCCTTAA-----T-----TTTGGATTAT--AAGATTAAATTACCTTAGG
GATAAC
>Garsauria aradoides
-----------TTTTAATTTATATTAAGTCTGGCCTGCTCAATGAATA-------TATTG
TTTTAATAGCCGCAGTATTTTGACTGTGCAAAGGTAGCATAATCATTTGTCTTTTAATTG
GAGGCTTGTATGAACGGTCGAATGAAATATAAACTTTCTTTATTATTTA--TTT-TATAA
TTTAATTTTTGAGTTAAAAAGCTTAAATAAGC-TATGGAGGACGAGAAGACCCTATAGAG
TTTTATATTTTTT-ATTA------------TTAATT-TATTAAT----AAAT--------
----------TAAATTAAA-TTA--ATTTTAAATTAATATTTTATTGGGGCAATGGGAAT
ATT-TTTATAATTATTCTTATT-TA---ATTA----TTATTAATT-----TGTAA----A
-TTT---AAGATCCTTTA-----T-----TATGGATTAT--AAGTTTAAATTACCTTAGG
GATAAC
>Cydnus aterrimus
----------TGATAATTTTATTTTAGGTCAGACCTGCTCAGTGATAT-------ATTAA
ATTAAATAGCCGCAGTATTTTGACTGTGCAAAGGTAGCATAATCATTTGTCTTTTAATTG
TGGGCTTGTATGAATGGTTTAATGAAATATAATCTTTCTTTGTGTTATA--TTA-AAGAA
CTTAATTTTTAAGTTAAAAAGCTTAAATATAA-G-TGGGGGACGAGAAGACCCTATAGAA
TTTTATATTGTTA-CTGT------------TATGGA-TTATTTA----TTAT--------
-----------GTATTAAT-TTG--TATTGGTAATAATATTTTGTTGGGGTGATAGGAAG
ATT-TGTTAAACTCTTTTAATT-AT---TAAT----TTATTTATT-----AGTAT----T
-TTT---AAGATCCTTAA-----T-----TTTGGATTAT--TAGATTAAATTACCTTAGG
GATAAC
>Dalcantha dilatata
------------TGATATTTATATTAGGTCTAGCCTGCCCACTGATAATT------TAAA

```
TTTAAAGGGCCGCAGTATTTTGACTGTGCGAAGGTAGCATAATCATTTGTCTTTTAATTG
GGGACTTGTATGAATGGTTGGATGAGACATTGACTTTCTTTATTATAA---TTT-ATGAA
TTTAATTTTTTAGTTAAAAAGCTAAAATGTTT-T-TATAGGACGAGAAGACCCTATAGAA
TTTTATTTAATTT---------------TGTAATAT-TTAAATT----GGATAA------
----------TTATTTATT-TAT--TACTTTTATATAAATTTTGTTGGGGCGATGTAGAA
ATT-TTATGAACTTTCTATTTT-T----TTTA----TTATGAATT-----AATAT----T
-TTA---TTGATCCTTTT-----T-----TAAGGATAAC--AAGATTAAATTACCTTAGG
GATAAC
>Katakadia caliginosa
--------TGAGTTTAATTTATATTAGGTCTTACCTGCTCAATGATAT--------TTTT
ATTAAATAGCCGCAGTATTTTGACTGTGCAAAGGTAGCATAATCAATTGTCTTTTAATTG
TAGGCTTGTATGAATGGTTTGACGAGATATTATCTTTCTTTGTGTTATT--ATT-TTGAA
TTTGATTATTGAGTTAAAAAGCTTAAATGTTT-T-TATAGGACGAGAAGACCCTATAGAA
ATTTATTAATTAT-TGTT------------TATTTC-TCTGTAA----TGGTTT------
----------TTGATTTGG----TGGTTTACATTATTAATTGTGTTGGGGTGACAGGGAA
ATT-TTATTAACTTTCTTTAAT-TA--TTTAA----TTATTGATG-----AGTAAATT-T
-TTA---ATGATCCTATT-----T-----TATGGATATT--AAGATTAATTTACCTTAGG
GATAAC
>Pseudoscoparipes fraterculus
----------TGTGTTGTATTTATTAGGTCAGGCCTGCTCAATGATTGTT-----TATGA
ATTTAATAGCCGCAGTATTTTGACTGTGCAAAGGTAGCATAATCATTTGTCTCTTAATTG
TGGGCTTGTATGAATGGTTTGACGAGACATTGGCTTTCTTTACATTATA--GTA-TTGAA
TTTAATTTTTGAGTTAAAAAGCTTAAATGGTT-T-TGTAGGACGAGAAGACCCTATAGAA
ATTTATTAATAAT-GTTTA-----------TCTTTTATGCTTTT----AGGTTA------
-----TTA--TAGAGTTTA-TGG--GGTATTATTATTAATTTTGTTGGGGTGACAGGGGG
ATT-ATTTTAACTCCCTTTAAA-TG--TTTAT----TTATTGATT-----TATAT----A
-TGT---GTGATCCTGTA-----T-----TATGGATAGT--AAGATTAATTTACCTTAGG
GATAAC
>Pseudoscoparipes vollenhoveni
---------TGTGTTTATTTATATTAGGTTAGGCCTGCTCAATGATTTATT----TATTG
ATTTAATAGCCGCAGTATTTTGACTGTGCAAAGGTAGCATAATCATTTGTCTTTTAATTG
TAGGCTTGTATGAATGGTTTGACGAGACATTGGCTTTCTTTGAACTATT--TTG-TTGAA
TTTAATTTTGAGTTAAAAAGCTTAAATGCTT-T-TGTAGGACGAGAAGACCCTATAGAA
ATTTATTAGTGTA-TATA------------TCTTT--TGCTTTT----AGATTA------
-----ATA--AAGTTTTAT-ATT--GATATTACTATTAATTGTGTTGGGGTGACAGGGTG
ATT-GTTATAACTCACTATGAT-TG--TTTAT----TTATTGATT-----TATAT----A
-TAT---TTGATCCTATT-----T-----TAAGGACAGT--AAGATTAATTTACCTTAGG
GATAAC
>Pseudoscoparipes kinabalensis
--------TGTTGGGTGTATTTATTAGGTCAGGCCTGCTCA-TGATTA--------ATTG
TTTAAATAGCCGCAGTATTTTGACTGTGCAAAGGTAGCATAATCATTTGTCTTTTAATTG
TAGGCTTGTATGAATGGTTTGACGAGACATTGACTTTCTTTGGGTTATTGATAA-TTGAA
TTTAATTTTTTAGTCAAAAAGCTAAGATGCTT-T-TATAGGACGAGAAGACCCTGTAGAA
ATTTATTAATTAT-ATTT------------TCTTTT-TCTTTTG----GGTTATGGAGTT
TTTATATG--AAGTTTTAA-TTT--GAGATTATAATTAATTGTGTTGGGGTGACAGGGAA
ATT-TTATTAACTTTCTTTAAT-TA---TTAT----TTATTAATT-----TGTAT----G
-TTA---GGGATCCTATT-----T-----TAAGGATAAT--AAGATTAATTTACCTTAGG
GATAAC
>Megymenum brevicorne
----------TTGGATATTTAAATCAGGTCTAACCTGCCCACTGAT---------AGTTA
ATTGAAGGGCCGCAGTAATTTGACTGTGCGAAGGTAGCATAATCATTTGTCTATTAATTG
TAGGCTTGTATGAATGGTTTGATGAGATACTGGCTTTCTTTATATAAAA---GG-ATGAA
TTTAATTTTTAAGTTAAAAAGCTTAAATAGTA-T-TGGAGGACGAGAAGACCCTATAGAA
TTTTATTATTATA-TTAT------------TTTATC-TTTTTTT----GGATGT------
```

-----TTA--ATGATTAAA-TTT--AAGATAATAAATAATTGTGTTGGGGCGATATGGAA
ATA-TTATGAACTTTCTTTATT-TA---TTAT----TTATTGATT-----TGTAT----T
-TTA---AGGATCCTCTT-----T-----TTGGGATTAT--AAGATTAAATTACCTTAGG
GATAAC
>Cyrtomenus emarginatus
---------TGGTTATATTTATTTCAGGTCTGGCCTGCTCAATGATTATT-----ATTTA
ATTAAATGGCCGCAGTACTTTGACTGTGCGAAGGTAGCATAATCATTTGTCTTTTAATTG
TAGGCTTGTATGAATGGTTTGATGAAGTACTAACTTTCTTTTGATCATA--AAA-AAGAA
CTTAATCTTTGAGTTAAAAAGCTTAAATTCTT-T-CATAGGACGAGAAGACCCTATAGAA
TTTTATTATAGGG-GTTA------------TATT---TTATGTG----AGGTTT------
----------TTATTAATA-GGA--TATATCTCTTATAATTTTGTTGGGGTGACAGGGAA
ATT-GTATAAACTTTCTTTTTATTT---GTTT----TTATTTATT-----TATAT----G
-TTA---TTGATCCCTTGA----T-----TAGGGATATT--AAGATTAAATTACCTTAGG
GATAAC
>Lactistes obesipes
--------TGAGGTTATAGTTAATCTGGTCAGTCTCGCTCATGATTGA-------ATTTA
ATTTAATAGCCGCAGTAATTTGACTGTGCGAAGGTAGCATAATCATTTGTCTATTAATTG
TAGACTTGTATGAAGGGCTTAATGAAATACTAACTTTCTTTTTTTTATA--AGA-GTGAA
TTTAATGTCTGAGTTAAAATGCTTAGATTTAT-T-TATAGGACGAGAAGACCCTATAGAA
TTTTATTATAAAT-TTGT------------TAAT---TTATTTT----ATGTAT------
---------TTTGATAATG-ATT--TGATGAATTTATAATTTTGTTGGGGCGACAGGGAA
ATT-TTATTAACTTTCTTTTAT-TT---TATT----TTAATTATT-----ATTAT----T
CCAA---TTGATCCTATA-----T-----TATGGATAGT--AAGATTAAATTACCTTAGG
GATAAC
>Lactistes vericulatus
--------TGGGTTTAACGTATATCAGGTCAAGTCTGCTCAATGATTT-------ATTTA
ATTTAATAGCCGCAGTAATTTGACTGTGCGAAGGTAGCATAATCATTTGTCTATTAATTG
TAGACTTGTATGAAGGGCTTAATGAAATACTAGCTTTCTTTTTTTTATA--TTA-GTGAA
CTTAATGTTTGAGTTAAAAGGCTTAAATTTAT-T-TATAGGACGAGAAGACCCTATAGAA
TTTTATTGTAATT-ATTA------------TTTAT--TTATTTA----TTGTAT------
----------TTTATAATA-AGT--TGGTTTTGTTACAATTTTGTTGGGGCGACAGGGAA
ATT-TTTTTAACTTTCTTTATTATT---TATT----TTAGTGATT-----ATTATTT--A
-TTA---TTGATCCTTTA-----T-----TATGGATAGT--AAGATTAAATTACCTTAGG
GATAAC
>Coptosoma bifarium
------------TGTTATTTATTTCAGGTTTGGCCTGCTCAATGATTT--------AATT
ATTAAATAGCCGCGGTATTTTGACCGTGCAAAGGTAGCATAATCAATTGTCTCTTAATTA
GAGACTTGTATGAATGGTTTGATGAGAAACTAACTTTCTTTATTATAAG--TTA-ATGAA
CTTAATTTTTAGGTTAAAAAGCTTAAATGTTT-C-TGTGGGACGAGAAGACCCTATAGAA
ATTTACTATCCCT-GTGA------------CACTTA----TTTA----TATTTA------
----------TTTAAAATA-AAT--GTTAGTAGTGATAGTTTTGTTGGGGCGACATGGAG
ATT-TTACAAACTCTCTACTTT-TA---ATTT----TTATAAGTG-----TGTAT----A
-TTA---TTGATCCTTTA-----T-----TAAGGATATT--GAGATTAATTTACCTTAGG
GATAAC
>Coptosoma scutellatum
-----------TGTTAATTTATAGCAGGTCTAGCCTGCTCAATGATTAGAAT---ATCTA
GTTAAATAGCCGCAGTATTTTGACTGTGCAAAGGTAGCATAATCAATTGTCTCTTAATTA
GAGGCTTGTATGAATGGTTTAATGAAAAATAAACTTTCTTT-TTAAAAA--TTG-ATGAA
TTTAATTTTTAGGTTAAAAAGCTTAAATGGTT-T-TGTGGGACGAGAAGACCCTATAGAA
ATTTACTATTGGT-GTAG------------TATTTA-TT-GTTA----TTTTAA------
----------TTTTAGATT-AAT--GCTTATTTCGATAGTTTTGTTGGGGCGACATAGAG
ATT-TTAAAAACTCTCTTTTTT-TG---TAAA----TTATAAATA-----CATAT----A
-TTA---TTGATCCTTTA-----T-----TAAGGATATT--AAGATTAATTTACCTTAGG
GATAAC

```
>Megacopta cribraria
----------TTGTTTATTTATTTTAGGTCTGGCCTGCTCAATGATTA--------TTAT
TTTAAATAGCCGCAGTATTTTGACTGTGCAAAGGTAGCATAATCATTTGTCTCTTAATTG
GGGGCTTGTATGAATGGTTTGATAAGGATTCGACTTTCTTTACTTAATA--TTA-ATGAA
TTTAATTTTTGGGTTAAAAAGCTCAAATATTT-T-TATGGGACGAGAAGACCCTATAGAA
ATTTACTCTATTT-TTGG------------TGTTTA----TTTT----TATTCT------
----------TTTTGGAAT-TTT--TATCATTACTAGAGTTTTGTTGGGGTGACATGAAG
ATT-GTTATAACTCTTTGTTAT-TA---CTTT----TTATTTATT-----TATAT----A
-CAA---CTGATCCTTTT-----T-----TATGGATTAA--AAGATTAATTTACCTTAGG
GATAAC
>Parastrachia japonensis
-------TGATTAATAATTTATTTTAAGTCTAACCTGCTCGATGATTA--------ATTT
ATTAAATGGCCGCAGTATTTTGACTGTGCAAAGGTAGCATAATCATTTGTCTTTTAATTT
GAGACTGGAATGAACGGTTGGACGAAATATTGACTTTCTTTACTTTATTATTTATATGAA
TTTAATATTTGAGTTAAAAAGCTTAAATATAT-A-AATAAGACGAGAAGACCCTATAGAA
TTTTATATAAAAA-TTTA------------TATTAT-GTTTTTT----GGGA--------
----------TTTGTTCA--TGT--ATTTAATTTTTGTATTTTGTTGGGGTGACAGGGAA
AAT-TATTTAACTTTCTTTTTA-TA---AATA----TTAATAATT-----ATTAT----A
-TTA---ACGATCCTTTA-----T-----TATGGATATT--AAGATTAAATTACCTTAGG
GATAAC
>Dolycoris baccarum
----------TGATTTAATTATACTAAGTCTGGCCTGCTCAATGATTT-----------T
TTTAAATAGCCGCAGTATATTGACTGTGCAAAGGTAGCATAATCATTTGTCTTTTAATTG
AAGGCTTGTATGAATGGTTGGATGAGGGAACGACTTTCTTTATATTAGG---AA-TTGAA
TTTAATTTTTTAGTTAAAAAGCTTAAATTTAT-A-AGTGGGACGAGAAGACCCTATAGAA
TTTTATTTTATTG-TAAT------------AATT---ACTATTT----TAGGGT------
----------TTAATTATT----AAATGTTACAATAAAATTTTGTTGGGGTGATGGTGAG
ATT-TTTATAACTCTCATAATT-TA--TATTT----TTATTAATT-----AGTAT----T
-TTT---AAGATCCTATT-----T-----TATGGATAAT--TAGTTTAAATTACCTTAGG
GATAAC
>Eurydema maracandica
----------TGTTATAATTATATTAAGTCTGGCCTGCTCAATGATTT---------TAT
ATTAAATAGCCGCAGTATTTTGACTGTGCGAAGGTAGCATAATCATTTGTCTTTTAATTG
AAGGCTTGTATGAATGGTTGGATGAGGGAATAGCTTTCTTTATATTAAA---TA-TTGAA
TTTAATTTTTTAGTTAAAAAGCTTAAATATTT-A-AATGGGACGAGAAGACCCTATAGAA
TTTTAATATTTAA-ATTA------------TTATTT-AATAATT----TTGGGT------
----------AATACTTT--TAT--TAATAATTTAATATTTTTATTGGGGTGATAGTGAG
ATT-TAATTAACTCTCATAATA-TA---ATTT----TTATTAATT-----AATAT----A
-TTT---AAGATCCTATA-----T-----TAAGGATAGT--TAGATTAAATTACCTTAGG
GATAAC
>Graphosoma italicum
----------TGTTTTTTTATTATTAAGTCTGGCCTGCTCAATGATTT-----------T
ATTAAATAGCCGCAGTATTTTGACTGTGCGAAGGTAGCATAATCATTTGTCTTTTAATTG
AAGGCTTGTATGAATGGTCGGATGAAAAAATGACTTTCTTTACATTAAA---TG-TTGAA
TTTAATTTTTTGGTTAAAAAGCTAAAATAATT-G-AATGGGACGAGAAGACCCTATAGAA
TTTTATTAAATTG-TAGT------------TTTGAA---TTTAT----TGGTTA------
----------ATTATTATT-TTT--AATTATAATTTTAATTTTGTTGGGGTGACATTGAG
ATT-TATTTAACTCTCATAATA-TA---AAGT----TTATTGATT-----TATAT----A
-TAA---AAGATCCTATT-----T-----TATGGATTAA--TAGATTAAATTACCTTAGG
GATAAC
>Picromerus griseus
-----------GGTGTATTTGTATTAAGTCTAGCCTGCTCAATGATTTTTATTATATAGG
ATTAAATGGCCGCAGTATTTTGACTGTGCGAAGGTAGCATAATCATTTGTCTTTTAATTG
AAGGCTTGCATGAATGGTTGGATGAGGGAATAGCTTTCTTTATATTAAA---AG-TTGAA
```

```
TTTAATGTTTCAGTTAAAAAGCTGAGATTTTT-A-AGTGGGACGAGAAGACCCTATAGAA
TTTTTACTAACAG-GGTT------------TAAGTA-TTATTTT----------------
----GATA--AGATCAAATACTT--ATATACTAGTTAAGTTTTGTTGGGGTGACAATGAG
ATT-TATTTAACTCTCAAATAT-AA--TTTAT----TTATTAATT-----TATAT----A
-TTT---AGGATCCTATT-----A-----TAAGGATAAT--TAGATTAAATTACCTTAGG
GATAAC
>Gonopsis affinis
-----------TGTTTTGGTTTATTAAGTCTGGCCTGCTCAATGATTA----------TT
ATTAAATAGCCGCAGTATTTTGACTGTGCGAAGGTAGCATAATCATTTGTCTTTTAATTG
GAGACTTGAATGAATGGTTTTATGAGGGAACGCCTTTCTTTATACTAAA---TT-TTGAA
TTTAATATTTGTGTTAAAAAGCGTAAATTTTT-A-AATGGGACGAGAAGACCCTATAGAA
TTTTATTTTATAT-TTAA------------TTATTT-GATCTAT----TGGTTT------
----------ATTATTAGA-TAT--AATTTAATTTAGAATTTTGTTGGGGCGACATTGAG
ATT-TTTTTTACTCTCATATAT-TA--TTTAT----TTATTAATT-----TTTAT----A
-TTT---AGGATCCTTTA-----T-----TATGGATATT--TAGTTTAAATTACCTTAGG
GATAAC
>Erthesina fullo
------------TGTTATATTTATTAAGTCTGGCCTGCTCAATGATTA---------TAA
ATTAAATAGCCGCGGTATTTTGACTGTGCAAAGGTAGCATAATCATTTGTCTTTTAATTG
AAGGCTTGAATGAATGGTTTGATGAGAAAATGACTTTCTTTACATTATG---GA-TTGAA
TTTAATGTTTTAGTTAAAAAGCTAAAATATAA-A-AGTGGGACGAGAAGACCCTATAGAA
TTTTATTTCTTAT-TGAT------------TATT---AATTTTT----TGGTTA------
----------ATATATTAT-TCT--TATTTAATAGGGAATTTTGTTGGGGTGACAGTGGG
ATT-TTTATAACTCCTATAATC-AA---ATAT----TTACATATC-----AGTAT----T
-TTA---AAGATCCTTT------T-----TTTTGATAAT--TAGATTAAATTACCTTAGG
GATAAC
>Alonips obsoletus
---------TAGAGTGATTTATTTTAGGTCGGGCCTGCTCAGTGATTA--------TTTG
ATTAAATAGCCGCAGTATTTTGACTGTGCGAAGGTAGCATAATCATTTGTCTTTTAATTG
TAGGCTTGTATGAAGGGTTTGATGAAATATTGACTTTCTTTGTATTAAA---TA-TATAA
CTTAATGATTTAGTTAAAAGGCTAAGATTTTT-T-CAGAGGACGAGAAGACCCTATAGAA
TTTTATTATTGCT-ATAA------------TTTTAT-TTATTTA----GGTACTGT----
----------TGGTATAAT-TGA--AGTTTTTAGGATAATTTTGTTGGGGCGACAGGGAA
ATT-TGATTAACTTTCTTTATT-TT---TATT----TAATAATTG-----TTATT----G
-TTA---GAGATCCTTTA-----T-----TAGGGATTAT--AAGATTAATTTACCTTAGG
GATAAC
>Cyclopelta obscura
-------TGTTAATTATTTTATTTTAGGTCTGTCCTGCCCTATGAGTT-------TATAT
CTTGAATGGCCGCGGTAATTTGACCGTGCGAAGGTAGCATAATCATTTGTCTATTAATTG
TAGGCTTGTATGAATGGTTTAATGTAATATTATCTTTCTTTGTTTTATA--ATT-GTGAA
CTTAATATTTTAGTGAAAAAGCTTAAATTTAT-T-TGGAGGACGAGAAGACCCTATAGAA
TTTTATTTACTGT-TTTT------------TAATCA----TTTT----TGGTAAA-----
----------TCAATATATGGTT--TAATATTTAGTAAATTTTGTTGGGGCGACATGGAA
ATT-TTTTTAACTTTCTGTATT-GA--TATTG----TTATTGATT-----TATAT----A
-CTA---AAGATCCTTTA-----T-----TATGGATTAT--TAGATTAAATTACCTTAGG
GATAAC
>Amnestus ficus
------------TAGTCTTTATTATAGGTTTGACCTGCTCAATGATTA---------TAA
ATTAAATAGCTGCAGTATTTTGACTGTACAAAGGTAGCATAATCATTTGTCTTTTAATTG
AAGGCTGGTATGAAGGGTCGGACGAGATACCTGCTTTCTTAATAATAA---TTT-ATGAA
TTTAAAATTTAGGTTAAAATGCTTAAATGTTT-T-TAAGGGACGAGAAGACCCTATAGAA
TTTAAATATAATT-TTAT------------ATT----TTATTTC----TGGTTA------
----------TTATTGTATTTAA--TTTTAAAATAATATTTTTATTGGGGTGATAGGAAA
ATT-TGTTTAACTTTTTTTATT-TA---TTTT----TCATGAATT-----AATGT----A
```

```
TTTA---AAGATCCCTTA-----A-----TAAGGATTAT--AAGATTAAATTACCTTAGG
GATAAC
>Amnestus zacki
------------TAGAATTTATTATAGGTTTAACCTGCTCAATGATTA---------ATA
ATTAAATAGCTGCAGTATTTTGACTGTACAAAGGTAGCATAATCATTTGTCTTTTAATTG
AAGGCTGGTATGAAGGGTTGGACGAAATACCTACTTTCTTAATAATAA---TTT-AAGAA
TTTAAAATTTAGGTTAAAATGCTTAAATTTTT-T-TAAGGGACGAGAAGACCCTATAGAA
TTTTAATTTTATT-TTAT------------GAT----TTGTTTT----TAGT--------
----------TTTTATATTTTAT--TTTTAAAATAATATTTTTGTTGGGGTGATAGGAAA
ATT-TTTATAACTTTTTTTATT-AA---TTTA----TCATTAATT-----AATGT----G
TTTA---ATGATCCTTTA-----T-----TAAGGATTAT--AAGATTAAATTACCTTAGG
GATAAC
>Lattinestus amplus
------------TTGTTTAAATATTAGGTTTAACCTGCTCAATGATTC----------AA
TTTAAATAGCTGCAGTATTTTGACTGTACAAAGGTAGCATAATCATTTGTCTTTTAATTG
AAGGCTAGTATGAAGGGTTGGACGAGGTATTATCTTTCTTGATAATATT---AT-TAGAA
TTTAAATTTTAAGTTAAAATGCTTAAATTTGT-T-TATGGGACGAGAAGACCCTATAGAA
TTTAATTTTATTT-TTAG------------GCGATAGATAACTT----TAGT--------
----------TATTTTATTATTT--TTCTTAAAATATAATTTTATTGGGGTGATAGGGAA
ATG-TAATTAACTTTTTTTATT-AA---TATT----TCATTGATT-----AATGT----T
TTTA---AAGATCCTTTA-----T-----TTAGGAGATA--AAGATTAAATTACCTTAGG
GATAAC
>Lattinestus barrerai
------------TTGTTATAATATTAGGTTTGACCTGCTCAATGGTTA----------AT
TTTAAATAGCTGCAGTATTTTGACTGTACAAAGGTAGCATAATCATTTGTCTTTTAATTG
AAGACTAGTATGAAGGGTTGGACGAGGTACTAACTTTCTTAATAATAAT---TA-TAGAA
TTTAAATTTTAAGTTAAAATGCTTAAATTTAT-T-TATGGGACGAGAAGACCCTATAGAA
TTTTATTTTATTT-TAAT------------TATATAATTAACAT----TAAT--------
----------TATTTTATA-TTT--TTTTTAAAATATAATTTTATTGGGGTGATAGGAAA
ATT-TATTAAACTTTTTTTATT-AA---AATT----TCATTAATG-----AGTGT----G
TTTA---ATGATCCTTAA-----TAAGTATTGGGATATG--AAGATTAAATTACCTTAGG
GATAAC
>Amnestus pusio
---------ATGTTTAAATATTATTAGGTATGACCTGCTCAATGATAA----------AT
TTTAAATGGCTGCAGTATATTGACTGTACAAAGGTAGCATAATCATTTGTCTTTTAATTG
GGGACTGGAATGAATGGTTGGACGAGATAATTACTTTCTTTATAATAAT--GTT--ATAA
TTTGAATTTTAAGTTAAAATGCTTAAATTTTT-T-TAAGGGACGATAAGACCCTATAGAA
TTTTATTTTTTTG-TATT------------ATAATAATTTATAT----------------
------TA--ATTTAAATT-ATA--TAATTTTAATGGAATTGTGTTGGGGTGATGGGAAA
ATT-TAATTAACTTTTTTTTTC-----ACTTA----TCATTAATG-----TATGT----A
TTTA---ATGATCCTTT------T-----TATGGATAAT--AAGATTAAATTACCTTAGG
GATAAC
>Nishadana umbrosa
-----------TATTTATTTGTTATAGGTCTAGCCTGCTCGGTGATATT------TTTAT
ATTAAACAGCCGCAGTATATTGACTGTGCAAAGGTAGCATAATCATTTGTCTCTTAAATG
GAGGCTGGTATGAATGGTTGGATGAAATATAAGCTTTCTTTGTCTTAAT--TTA-TTGAA
TTTAATATTTCAGTCAAAAAGCTGAGATGATT-T-TGGGGGACGAGAAGACCCTATAGAG
TTTTACATTATTA-ATTA------------TATAC--AGACTTT----TGGTTT------
----------TATATTGTC-TTA--TATAATACTTGGTGTTTTGTTGGGGCGACAGGGGA
ATT-ATCATAACTTCTCTTAAT-AA---TTTT----TTACTGATT-----AGTAA----A
TTTA---TTGATCCTTCT-----T-----TGTGGATTAT--AAGATTAAATTACCTTAGG
GATAAC
>Peltoxys sataranus
TGT----CCTATATTTATATATTGTAGGTATAACCTGCTCAGTGATTT------------
```

ATTAAACAGCCGCAGTATACTGACTGTGCAAAGGTAGCATAATCATTTGTCTTTTAATTG
AAGGCTGGTATGAATGGTTGGATGAAATATAAGCTTTCTTTACTTTAT---TTG-TTGAA
TTTAATCTTTTAGTTAAAAAGCTAAAATTTTT-T-TGTAGGACGAGAAGACCCTATAGAG
TTTTATTTATTGA-TAAT------------TTAAT--CTAAGGT----TGGTTA------
----------ATTACTTAT----TTATTTATTGATAAATTATGTTGGGGCGACAGGGGA
ATT-TTTTTAACTTCCCTTAAT-TA---TAAA----TTATTGATT-----AATAA----T
TTTT---ATTATCCTGTA-----T-----TATGGATTAT--AAGATTAAATTACCTTAGG
GATAAC
>Stibaropus indonesicus
-----------CAGTCAATTATTTTACGTCTGGCCTGCTCAATGATTATTT----ATATA
ATTAAATAGCCGCAGTATCCTGACTGTGCGAAGGTAGCATAATCATTTGTCTTTTAATTG
TAGGCTAGTATGAATGGTCGGATGAGGCATCAACTTTCTTTATCTTATA--ATT--TGAA
TTTAAAGTTTGAGTTAAAAGGCTTAAATTTAT-T-CGTGGGACGAGAAGACCCTATAGAA
TTTTATTGTAATT-GTGA------------TTTTAT-CCATTTT----TGGTTGATA---
----------TTTGGTGGT-ATG--GAGTTGTATTGCAATTGTGTTGGGGCGACAGGGAA
ATT-AATGTAACTTTCTTTCATTTA---TTTA----TTACTGATT-----AGTAT----A
TTTA---AAGATCCTGTA-----T-----TATGGATTTA--AAGATTAAATTACCTTAGG
GATAAC
>Stibaropus molginus
-----------TTGTTAATTATTTTAAGTCTGGCCTGCTCAATGATTATTT----ATGTA
ATTAAATAGCCGCAGTATCCTGACTGTGCGAAGGTAGCATAATCATTTGTCTTTTAATTG
TAGGCTAGTATGAATGGTCGGATGAGGCATCAACTTTCTTTATCTTATA--ATT--TGAA
TTTAAAGTTTGAGTCAGAAGGCTTAAATTTAT-T-CGTGGGACGAGAAGACCCTATAGAA
TTTTATTGTAATT-GTGA------------TTTTAT-CCATTTT----TGGTTGATA---
----------CTTGGTGGT-ATA--GAGTTATATTGTAACTGTGTTGGGGCGACAGGGAA
ATT-AATGTAACTTTCTTTCATTCG---TTTA----TTATTGATT-----AGTAT----A
TTTA---AAGATCCTGTA-----T-----TATGGATTTA--AAGATTAAATTACCTTATG
GATAAC
>Microporus pallidipennis
----------TTGTGTAGTTATATTAAGTCAGGCCTGCTCAATGATTA-------GTTTA
ATTAAATAGCCGCAGTATTTTGACTGTGCGAAGGTAGCATAATCATTTGTCTTTTAATTG
TAGGCTAGTATGAAGGGTTTGATGAAGTTTATACTTTCTTTGTTTTAAT--ATT-AATAA
TTTAATGTTTAAGTTAAAAGGCTTAAATTTAT-T-CGGAGGACGAGAAGACCCTATAGAA
ATTTTTATTATTA-CTTAG-----------TAAAGT-TTACTTT----TGGTTT------
---------TATGTTGTTTAATC--TTTATTATTAATAATTGTGTTGGGGTGACAGAGGG
GTT-TTTTTAACCCCCTTTATT-AA-TTATTA----TTGTTGATT-----ATCAT----G
TTTA---TTGATCCTTGG-T--TT-----CGAAGATTAT--AAGATTAATTTACCTTAGG
GATAAC
>Thaumastella elizabethae
---------------TATTATTATCAGGTTTAACCTGCCCACTGATTT--------AATT
ATTTAAAGGCCGCAGTATATTAACTGTGCAAAGGTAGCATAATCAATTGTCTTTTAATTG
AAGGCTAGAATGAATGGTTGAATGAGAGATAGACTTTCTTTGCTATAT---TTA-TTGAA
TTTAATTTTTTAGTTAAAAAGCTAAAATTTGT-T-TAAAGGACGAGAAGACCCTATAGAA
TTTTACTCATATA-GATG------------AATTAT-TTATTAT----------------
----ATTA--GGTTATGTA-ATG--TTTATTTTATTGAGTTTTGTTGGGGTGACAAGGAA
AATGTTTATAACTTTCTTTATT-TA--TAATA----TTATTTATA-----TATAT----A
-TTA---GAGATCCTTAT-----T-----TTAAGATATA--AAGATTAAATTACCTTAGG
GATAAC
>Thaumastella namaquensis
-----------TGTTGGTTAATATTAGGTCTGGCCTGCCCACTGATTT-------AGTAG
ATTAAAGGGCCGCAGTATACTGACTGTGCAAAGGTAGCATAATCAATTGTCTTTTAATTG
AAGGCTGGTATGAATGGTTGGATGAAATGCATGCTTTCTTTATTTAATG--TTA-TTGAA
TTTAATTTTTAAGTTAAAAAGCTTAAATTTTG-T-TGAAGGACGAGAAGACCCTATAGAA
TTTTATCTAACTT-GGTT------------TTAGTA-TTTAAAG----------------

----------GTTGATTTT-ATG--TGAATTGAGTTGGATTTTGTTGGGGTGATGGGGAA
ATT-TATATAACTTTCTTTTTA-TA---AATA----TTATTTGTG-----AGTAT----A
TTTA---AAGATCTTTAT-----T-----TTAGGATAAT--AAGATTAAATTACCTTAGG
GATAAC
>Urochela quadrinotata
-----------ATGAATTAAATATAAGGTCAGGCCTGCTCGGTGATTT------------
ATTTAACAGCTGCAGTATATTGACTGTGCAAAGGTAGCATAATCATTTGTCTTTTAATTG
GGGACTAGTATGAACGGTTTGATGAAATATTAACTTTCTTTTTATATTA--ATT-TTGAA
ATTAATTTTTGTGTTAAAAAGCATAAATTCAA-T-TATAGGACGAGAAGACCCTATAGAA
TTTTGAATATCCA-TATT------------TTTATA-GTAGATT----------------
------TA--TTATCAAGTATTT--AAAAAATAGTATATTTTTGTTGGGGTGACAGAGGA
ATA-TATCAAACTTCCATTTAA-AT---AATT----TTATAAATT-----TATAT----A
-TTA---TTGATCCTAT------T-----TTTAGAATAT--AAGATTAAATTACCTTAGG
GATAAC
>Urostylis flavoannulata
-------GTGATACATATTTATATTAGGTCGGGCCTGCTCAGTGATTT------------
ATTTAACAGCTGCAGTAATTTGACTGTGCAAAGGTAGCATAATCATTTGTCTTTTAATTG
AAGGCTGGTATGAATGGTTTGATGAAATATTAACTTTCTTTTTATTTAA--TTT-TTGAA
TTTAATTTTTATGTGAAAAAGCATAGATTATG-T-TGTAGGACGAGAAGACCCTATAGAA
TTTTAATTTAGTT-TATT------------TTATA--TTATTTA----TGGATA------
----------TTATAAATC-TTT--AATATTAATTATATTTTTGTTGGGGTGACAGGAAA
ATT-TATTTAACTTTTTCTTTT-TA---ATTT----TTATTCTTC-----CATAT----A
-TTA---ATTATCCTAA------T-----TATAGATTAC--AAGATTAAATTACCTTAGG
GATAAC
>Teabooma secunda
--------TTGTTGAGGTTTATTTCAGGTCAGGCCTGCTCAGTGATTTATATGG-AGATA
ATTAAATAGCCGCAGTAATTTGACTGTGCAAAGGTAGCATAATCATTTGTCTTTTAATTG
TAGGCTTGTATGAATGGTTTAATGAATCATTAACTTTCTTTAAACTAAT-ATTT-TAGAA
TTTAATTTTTGAGTTAAAAGGCTCAGATCTAT-T-CGTAGGACGAGAAGACCCTATAGAA
TTTTATTTTATTG-AGAT------------ACAGTC-AGGCTTT----TGGTAAAAA---
------TA--GTGGCTAATAAGT-TTTATTAATAAAAGTTGTTGTTGGGGCGATGGGGAA
ATT-TATTTAACTCTCTTTAAT-AA---TTAT----AAATGTATT-----ATTAT----T
----------TTCCTTTA-----T-----TAAGGATAAT--AAGATTAAATTACCTTAGG
GATAAC
>Libiocoris heissi
-------------TGGTTTTTTTTTAAATCTCGCCTGCTCAATGATTAT-----------
TTTAAATAGCTGCAGTATTTTGACTGTACAAAGGTAGCATAATAATTTGTCTTTTAATTG
AAGGCTTGAATGAATGGTAGGACGAGGGGCAAGCTTTCTTTAAACTATT--TTA-TTGAA
TTTAATTTTTTAGTTAAAAAGCTGAGATTTTA-T-TGTAGGACGAGAAGACCCTGTAGAA
TTTTACCTCTATT-TGTT------------TTTATT-ATTTAT-----------------
---------TTTAAATGTTATTT--TAATGAAGGTTAGGTTTTGTTGGGGAGACAGAGTT
TTT-TTATTAACGAACTTTTGT-TT---ATTT----TCATTGATT-----ATTGT----T
GTTA---ATGATCCTTTA-----T-----TATGGATTGT--AAGATTAAATTACCTCAGG
GATAAC
>Himacerus mirmicoides
---------TGAGTTTATTAATATGAGGTCTGACCTGCCCTATGATTTA------TATGA
ATTAAATGGCCGCAGTATTCTAACTGTGCGAAGGTAGCATAATCATTTGTCCTTTAATTG
GGGACTTGTATGAATGGTCTGATGAGGGATAAACTTTCTTTATTTTATT--TTT-AATAA
TTTAATTTATAAGTCAAAAAGCTTATATATTA-T-TAAGTGACGAGAAGACCCTATAGAA
CTTTACTATTTAT-AATG------------ATACT--GTTATTT----TGTTTA------
----------TTAATTGAT-AGT--ATTTTTATTAGTAGTTTTATTGGGGCGATAGTTAA
ATT-TAATTAACTTAAACTTGATTA---AATT----TCATTAATT-----AATGATTA-A
-TTA---TTGATCCATTT-----T-----TATTGATTAA--AAGATCAAGTTACCTTAGG
GATAAC

```
>Nabis apicalis
----------TGTATACTTAATATAAGGTCTGACCTGCCCAATGATTA-----------T
ATTTAATGGCCGCAGTATATTGACTGTGCGAAGGTAGCATAATCACTTGTCCTTTAATTG
TGGACTAGTATGAATGGTTTAATGAGGGATATACTTTCTTTATTTTATA--TAT-ATAAA
TTTAATTTATAAGTTAAAAAGCTTATATAATT-T-TAAATGACGAGAAGACCCTATAGAA
CTTTACTTATTAT-ACCT------------ATTGT--AATTATT----TGTTTT------
----------GTTATAAAT-ATA--GTCTGTAACATAAGTTTTGTTGGGGTGACAATTAA
ATC-TTTTTAACTTTAATTTAAATA--TTAAA----TCATTAATT-----AATGTAT--A
TTAA---TTGATCCTGTA-----A-----TATGGATTAT--AAGAATAAGTTACCTTAGG
GATAAC
>Canthophorus impressus
-------TGATAAAATATTGATTTTAGGTCAGGCCTGCTCAGTGATTAT------AAAAA
ATTAAATAGCCGCAGTATTTTGACTGTGCAAAGGTAGCATAATCATTTGTCTATTAATTG
TAGGCTAGTATGAATGGTTTGATGAGATATTAACTTTCTTTATATATTT--TTT--ATAA
ATTAATATATTAA--ATATCCTTTAATTATGG-A-AATAAGATTAAATTACCTTAGGGAT
AACAGCGTAATTC-TTTT------------GGAGAG-TTCATAT----CGATAA------
----------AAGATTAAA----AAATTATGTAATTAAATTTTGTTGGGGTGATAGGGAA
AAT-TTCATAACTTTCTTTATA-TA---TTTG----TTATAAATT-----AATAT----A
-TTA---AATATCCTTTA-----T-----TAAGGA-AAT--AAGATTAAATTACCTTAGG
GATAAC
>Corythucha ciliata
-------------TGTTTTTATAAAAAATATAACCTGCCCTATGATT---------AAAA
GTTAAATGGCCGCGA---TTTTATCGTGCAAAGGTAGCATAATAATTTGTCCTTTAAATG
AGGTCTAGAATGAAAGGTTGAACGAGAGAGTAGCTTTCTTATTTTAATT--TTA-TTGAA
TTTTATTTTTTGGTTAAAAAGCTAAAATGAAT-T-TATGGGACGATAAGACCCTATAGAA
TTTAATTAACCTT-ATAA------------GATATA-GTTTTTA----------------
--------TTTTTAATTTT-ATA--TCTTATAAGGTTAATTTAGTTGGGGCGACTATTAT
ATT-TGATATACTATATTTTTA-TA---TTTT----TCATTGTTT-----TATGA----A
-AAT---TTGATCCTGAA-----T-----TTTAGATTAT--AAGAATAAATTACCTTAGG
GATAAC
```
