# Supplementary material for: New Molecular Phylogenetic Evidence Confirms Independent Origin of Coxal Combs in the Families of the ‘Cydnoid’ Complex (Hemiptera: Heteroptera: Pentatomoidea)
Source: Insects. 2024 Oct 11;15(10):792. doi: 10.3390/insects15100792 (PMC11509079; doi:10.3390/insects15100792)
Supplement: Supplementary file 1 [file insects-15-00792-s001.zip › Table S1.pdf]

Table S1. List of specimens used in the phylogenetic analysis, their geographic origin (if provided), GenBank accession numbers, and the sources for the sequences downloaded from GenBank (all taxa listed in alphabetical order).

**Infraorder**	**Superfamily**	**Family**	**Species**	**Geographic origin (year collected, if originally provided)**	**GenBank accesion numbers for 16S rDNA**	**Source**
Pentatomomorpha	Aradoidea	Aradidae	*Libiocoris heissi* Bai, Yang & Cai 2006	China	JQ780819	Song et al. 2016
	Coreoidea	Alydidae	*Leptocorisa acuta* (Thunberg, 1783)	No data	AY252691	Wheeler & Schuh Direct submission to GenBank (2003)
			Riptortus pedestris (Fabricius, 1775)	China	EU427344	Hua et al. 2008
		Coreidae	*Cletus punctiger* (Dallas, 1852)	China (2010)	AY986810	Li Direct submission to GenBank (2007)
		Rhopalidae	*Stictopleurus subviridis* Hsiao, 1977	China	EU826088	Hua et al. 2009
		Stenocephalidae	*Dicranocephalus femoralis* (Reuter, 1888)	China (2011)	JQ910990	Li et al. 2017
	Lygaeoidea	Geocoridae	*Geocoris pallidipennis* (A. Costa, 1843)	China	EU427336	Hua et al. 2008
		Lygaeidae	*Kleidocerys resedae* (Panzer, 1797)	China	KJ584365	Li et al. 2016
			Lygaeus equestris (Linnaeus,	Czech Republic (2010)	JQ234972	Lis et al. 2011
		Rhyparochromidae	*Neolethaeus assamensis* (Distant, 1901)	No data	KX505856	Jiang, Li & Cai Direct submission to GenBank (2018)
	Pentatomoidea	Acanthosomatidae	*Acanthosoma nigrodorsum* Hsiao & Liu, 1977	China	LC099172	Tsai et al. 2015
			Elasmostethus interstinctus (Linnaeus, 1758)	Poland (2007)	JQ029152	Lis et al. 2012
			Elasmucha grisea (Linnaeus, 1758)	Poland (2009)	JQ029151	Lis et al. 2012
			Elasmucha laeviventris Liu, 1919	China (2011)	LC099199	Tsai et al. 2015
			Lindbergicoris hochii (Yang)	China (2011)	LC099164	Tsai et al. 2015
			Sastragala scutellata (Scott, 1874)	Japan	LC099181	Tsai et al. 2015
		Cydnidae	*Adomerus biguttatus* (Linnaeus, 1758)	Poland (2010)	PP357091	present paper
			Adomerus rotundus Hsiao, 1977	Japan (2010)	PP357092	present paper
			Adomerus triguttulus (Motschulsky, 1866)	Japan (2010)	PP357093	present paper
			Adomerus variegatus (Signoret, 1884)	Japan (2010)	PP357094	present paper
			Adrisa birmana J.A. Lis, 1992	Thailand (2013)	PP357095	present paper
			Adrisa magna (Uhler, 1861)	Japan (2010)	PP357096	present paper
			Adrisa romani J.A. Lis, 1994	Thailand (2013)	PP357097	present paper
			Aethus pseudindicus J.A. Lis, 1993	Vietnam (2013)	PP357098	present paper
			Alonips obsoletus Signoret, 1881	Australia (2018)	PP357099	present paper
			Amaurocoris curtus (Brullé, 1838)	Cyprus (2015)	PP357100	present paper
			Amnestus ficus Mayorga & Cervantes, 2001	Mexico (2014)	PP357101	present paper

			Amnestus pusio (Stål, 1860)	Guatemala (2011)	PP357102	present paper
			Amnestus zacki Mayorga & Cervantes, 2009	Guatemala (2010)	PP357103	present paper
			Blaena setosa Walker, 1868	Australia (2012)	PP357104	present paper
			Byrsinus pseudosyriacus (Linnavuori, 1977)	Namibia (2012)	PP357105	present paper
			Byrsinus varians (Fabricius, 1803)	Guam (2004)	PP357106	present paper
			Canthophorus impressus Horváth, 1881)	Poland (2010)	PP357107	present paper
			Canthophorus niveimarginatus Scott, 1874	Japan (2010)	PP357108	present paper
			Chilocoris confusus Horváth, 1919	Japan (2011)	PP357109	present paper
			Chilocoris neozealandicus Lariviére & Froeschner, 1994	Australia (1990)	PP357110	present paper
			Chilocoris capensis J.A. Lis, B. Lis & Compton, 2016	Republic of South Africa (2013)	PP357111	present paper
			Crocistethus waltlianus (Fieber, 1837)	Morocco (2002)	PP357112	present paper
			Cydnus aterrimus (Forster, 1771)	Poland (2010)	PP357113	present paper
			Cyrtomenus emarginatus Stål, 1862	Guatemala (2011)	PP357114	present paper
			Fromundus difficilis (Stål, 1854)	Namibia (2012)	PP357115	present paper
			Fromundus pygmaeus (Dallas, 1851)	Guam (2004)	PP357116	present paper
			Garsauria aradoides Walker, 1868	Brunei (2014)	PP357117	present paper
			Geotomus convexus Hsiao, 1977	Japan (2011)	PP357118	present paper
			Katakadia caliginosa (Walker, 1867)	Brunei (2014)	PP357119	present paper
			Lactistes obesipes Signoret, 1879	Australia (2018)	PP357120	present paper
			Lactistes vericulatus Schiødte, 1848	Zambia (2014)	PP357121	present paper
			Lattinestus amplus Eger, 2008	Costa Rica (1995)	PP357122	present paper
			Lattinestus barrerai Mayorga & Brailovsky, 2012	Guatemala (2007)	PP357123	present paper
			Legnotus limbosus (Geoffroy, 1785)	Poland (2014)	PP357124	present paper
			Macroscytus annulipoides J.A. Lis, 1999	Australia (2018)	PP357125	present paper
			Macroscytus badius (Walker, 1867)	India (2013)	PP357126	present paper
			Macroscytus brunneus (Fabricius, 1803)	Greece (2007)	JQ029138	Lis et al. 2012
			Macroscytus fraterculus Horváth, 1919	Japan (2010)	PP357127	present paper
			Macroscytus japonensis Scott, 1874	Vietnam (2013)	PP357128	present paper
			Macroscytus minimus J.A. Lis, 1999	Australia (2018)	PP357129	present paper
			Microporus nigrita (Fabricius, 1794)	Poland (2010)	JQ029137	Lis et al. 2012
			Microporus pallidipennis (Reuter, 1883)	Namibia (2012)	PP357130	present paper
			Nishadana umbrosa Horváth, 1919	India (2005)	PP357131	present paper
			Ochetostethomorpha secunda J.A. Lis & B. Lis, 2014	Namibia (2012)	PP357132	present paper
			Ochetostethus brachyscytus Reuter, 1891	Egypt (1997)	PP357133	present paper
			Ochetostethus heissi Magnien, 2006	Cyprus (2015)	PP357134	present paper
			Ochetostethus nanus Herrich-Schaeffer, 1834	Spain (1988)	PP357135	present paper
			Ochetostethus opacus (Scholtz, 1847)	Poland (2016)	PP357136	present paper
			Pangaeus bilineatus (Say, 1825)	Guatemala (2011)	PP357137	present paper
			Pangaeus rugiceps Horváth, 1919	Guatemala (2011)	PP357138	present paper
			Parachilocoris minutus (Distant, 1901)	Japan (2011)	PP357139	present paper

			Peltoxys sataranus J. A. Lis & B. Lis, 2007	India (2005)	PP357140	present paper
			Pseudoscoparipes fraterculus J.A. Lis, 1994	Thailand (2013)	PP357141	present paper
			Pseudoscoparipes kinabalensis J.A. Lis, 1994	Brunei (2015)	PP357142	present paper
			Pseudoscoparipes vollenhoveni (Signoret, 1881)	Brunei (2015)	PP357143	present paper
			Rhytidoporus indentatus Uhler, 1877	Guam (2004)	PP357144	present paper
			Sehirus luctuosus Mulsant et Rey, 1866	Poland (2010)	JQ234973	Lis et al. 2011
			Stibaropus indonesicus J.A. Lis, 1991	Brunei (2014)	PP357145	present paper
			Stibaropus molginus (Schiødte, 1848)	Thailand (1995)	PP357146	present paper
			Teabooma secunda J.A. Lis et B. Lis, 2010	New Caledonia (2004)	PP357147	present paper
			Tritomegas bicolor (Linnaeus, 1758)	Poland (2011)	PP357148	present paper
			Tritomegas sexmaculatus (Rambur, 1839)	Poland (2010)	PP357149	present paper
		Dinidoridae	*Cyclopelta obscura* (Lepeletier & Serville, 1828)	Thailand (1995)	JQ029146	Lis et al. 2012
			Megymenum brevicorne (Fabricius, 1787)	Thailand (1995)	JQ029141	Lis et al. 2012
		Parastrachiidae	*Dismegistus sanguineus* (DeGeer, 1778)	South Africa	EF641131	Grazia et al. 2008
			Parastrachia japonensis (Scott, 1880)	Japan (2001)	JQ029136	Lis et al. 2012
		Pentatomidae	*Dolycoris baccarum* (Linnaeus)	Poland (2009)	JQ029153	Lis et al. 2012
			Erthesina fullo (Thunberg, 1783)	China	AY986801	Li Direct submission to GenBank (2007)
			Eurydema maracandica Oshanin, 1871	China (2011)	MF135553	Zhao W. et al. 2017
			Gonopsis affinis (Uhler, 1860)	China (2015)	MG182695	Chen et al. 2017
			Graphosoma italicum (O.F. Müller, 1766)	Czech Republic (2011)	PP357151	present paper
			Picromerus griseus (Dallas, 1851)	China (2015)	MF805778	Zhao Q. et al. 2017
		Plataspidae	*Coptosoma bifarium* Montandon, 1897	China	EU427334	Hua et al. 2008
			Coptosoma scutellatum (Geoffroy, 1785)	Poland (2010)	JQ029144	Lis et al. 2012
			Megacopta cribraria (Fabricius, 1798)	No data	JF288758	Eaton & Jenkins Direct submission to GenBank (2012)
		Scutelleridae	*Eurygaster maura* (Linnaeus)	Poland (2002)	JQ029150	Lis et al. 2012
			Odontoscelis fuliginosa (Linnaeus)	Poland (2007)	JQ029149	Lis et al. 2012
			Poecilocoris nepalensis (Herrich-Schaeffer, 1837)	China (2009)	JQ743675	Li et al. 2017
		Tessaratomidae	*Dalacantha dilatata* Amyot & Serville, 1843	China (2009)	JQ910981	Li et al. 2017
			Eusthenes cupreus (Westwood, 1837)	China (2009)	JQ910983	Song 2013
		Thaumastellidae	*Thaumastella elizabethae* Jacobs, 1989	South Africa	EF641147	Grazia et al. 2008
			Thaumastella namaquensis Schaefer & Wilcox, 1971	South Africa	EF641148	Grazia et al. 2008
		Thyreocoridae	*Galgupha australis* McAtee & Malloch, 1933	Bolivia (2010)	PP357150	present paper
			Galgupha difficilis (Breddin)	Brasil (1975)	JQ029145	Lis et al. 2012
			Thyreocoris scarabaeoides (Linnaeus, 1758)	Poland (2009)	JQ029135	Lis et al. 2012
		Urostylididae	*Urochela quadrinotata* (Reuter, 1881)	China	JQ743678	Dai et al. 2012
			Urostylis flavoannulata Stål, 1854	China (2015)	KY069970	Jiang, Li &. Cai

						Direct submission to GenBank (2018)
	Pyrrhocoroidea	Largidae	*Physopelta gutta* (Burmeister, 1834)	China	EU427343	Hua et al. 2008
		Pyrrhocoridae	*Dysdercus cingulatus* (Fabricius, 1775)	China	EU427335	Hua et al. 2008
			Pyrrhocoris apterus (Linnaeus, 1758)	France	KX821818	Crumière et al. 2016
Cimicomorpha (outgroup)	Miroidea	Miridae (outgroup)	*Adelphocoris lineolatus* (Goeze, 1778)	No data	AY252764	Wheeler,W.C. and Schuh,R.T., unpublished
			Lygus hesperus Knight, 1917	USA	KF679984	Roehrdanz,R. and Cameron,S. unpublished
		Tingidae (outgroup)	*Corythucha ciliata* (Say, 1832)	China (2011)	KC756280	Yang et al 2013
	Naboidea	Nabidae (outgroup)	*Himacerus mirmicoides* (O. G. Costa, 1834)	Poland (2010)	PP357152	present paper
			Nabis apicalis Matsumura, 1913	China (2005)	EF487292	Tian et al. 2008

References

Chen, Ch., Wei, J., Ji, W., Zhao, Q. (2017) The first complete mitochondrial genome from the subfamily Phyllocephalinae (Heteroptera: Pentatomidae) and its phylogenetic analysis, Mitochondrial DNA Part B, 2:2, 938-939, DOI: 10.1080/23802359.2017.1413313

Crumière, A. J. J., Santos, M. E., Sémon, M., Armisén, D., Moreira, F. F. F., & Khila, A. (2016). Diversity in Morphology and Locomotory Behavior Is Associated with Niche Expansion in the Semi-aquatic Bugs. *Current biology : CB*, *26*(24), 3336–3342. https://doi.org/10.1016/j.cub.2016.09.061

Dai Y.T., Li H., Jiang P., Song F., Ye Z., Yuang X., Dai X., Chang J., Cai W.Z. Sequence and organization of the mitochondrial genome of an urostylidid bug, *Urochela. quadrinotata* Reuter (Hemiptera: Urostylididae) *Entomotaxonomia*. 2012;34:613–623.

Grazia J, Schuh RT, Wheeler WC (2008) Phylogenetic relationships of family groups in Pentatomoidea based on morphology and DNA sequences (Insecta: Heteroptera). *Cladistics* 24: 1–45.

Hua JM, Dong PZ, Li M, Cui Y, Zhu WB, Xie Q, Bu WJ. (2009) The analysis of mitochondrial genome of *Stictopleurus subviridis* Hsiao (Insecta: Hemiptera-Heteroptera: Rhopalidae) Acta Zootax Sin., 34:1–9.

Hua,J., Li,M., Dong,P., Cui,Y., Xie,Q. and Bu,W. (2008) Comparative and phylogenomic studies on the mitochondrial genomes of Pentatomomorpha (Insecta: Hemiptera: Heteroptera. BMC Genomics 9, 610.

Li, T., Yi, W., Zhang, H., Xie, Q., & Bu, W. (2016). Complete mitochondrial genome of the birch catkin bug Kleidocerys resedae resedae, as the first representative from the family Lygaeidae (Hemiptera: Heteroptera: Lygaeoidea). *Mitochondrial DNA. Part A, DNA mapping, sequencing, and analysis*, *27*(1), 618–619. https://doi.org/10.3109/19401736.2014.908372

Li, H., Leavengood, J. M., Jr, Chapman, E. G., Burkhardt, D., Song, F., Jiang, P., Liu, J., Zhou, X., & Cai, W. (2017). Mitochondrial phylogenomics of Hemiptera reveals adaptive innovations driving the diversification of true bugs. *Proceedings. Biological sciences*, *284*(1862), 20171223. https://doi.org/10.1098/rspb.2017.1223

Lis,J.A., Lis,P.A., Ziaja,D.J. (2011). Comparative studies on 12S and 16S mitochondrial rDNA sequences in pentatomomorphan bugs (Hemiptera: Heteroptera: Pentatomomorpha. *Nat J (Opole)* 44, 73-91 (2011)

Lis JA, Lis P, Ziaja DJ, Kocorek A (2012). Systematic position of Dinidoridae within the superfamily Pentatomoidea (Hemiptera: Heteroptera) revealed by the Bayesian phylogenetic analysis of the mitochondrial 12S and 16S rDNA sequences. *Zootaxa* 3423: 61–68.

Roehrdanz, R., Cameron, S.L., Toutges, M., Wichmann, S.S. (2016) The complete mitochondrial genome of the tarnished plant bug, *Lygus lineolaris* (Heteroptera: Miridae), *Mitochondrial DNA Part A*, 27:1, 48-49, DOI: 10.3109/19401736.2013.869689

Song, W., Li, H., Song, F., Liu, L., Wang, P., Xun, H., Dai, X., Chang, J., & Cai, W. (2013). The complete mitochondrial genome of a tessaratomid bug, Eusthenes cupreus (Hemiptera: Heteroptera: Pentatomomorpha: Tessaratomidae). *Zootaxa*, *3620*, 260–272. https://doi.org/10.11646/zootaxa.3620.2.4

Song, F., Li, H., Shao, R. *et al*. Rearrangement of mitochondrial tRNA genes in flat bugs (Hemiptera: Aradidae). *Sci Rep* **6**, 25725 (2016). https://doi.org/10.1038/srep25725

Tian Y, Zhu W, Li M, Xie Q, Bu W. Influence of data conflict and molecular phylogeny of major clades in Cimicomorphan true bugs (Insecta: Hemiptera: Heteroptera). *Mol Phylogenet Evol*. 2008;47(2):581-597. doi:10.1016/j.ympev.2008.01.034

Tsai JF, Kudo S, Yoshizawa K. Maternal care in Acanthosomatinae (Insecta: Heteroptera: Acanthosomatidae)--correlated evolution with morphological change. BMC Evol Biol. 2015 Nov 19;15:258. doi: 10.1186/s12862-015-0537-4. PMID: 26586480; PMCID: PMC4653913.

Yang W, Yu W, Du Y. The complete mitochondrial genome of the sycamore lace bug Corythucha ciliata (Hemiptera: Tingidae). *Gene*. 2013;532(1):27-40. doi:10.1016/j.gene.2013.08.087

Zhao, W., Zhao, Q., Li, M., Wei, J., Zhang, X., Zhang, H. (2017) Characterization of the complete mitochondrial genome and phylogenetic implications for *Eurydema maracandica* (Hemiptera: Pentatomidae), *Mitochondrial DNA Part B*, 2:2, 550-551, DOI: 10.1080/23802359.2017.1365649

Zhao, Q., Wei, J., Zhao, W., Cai, B., Du, X., & Zhang, H. (2017). The first mitochondrial genome for the subfamily Asopinae (Heteroptera: Pentatomidae) and its phylogenetic implications. *Mitochondrial DNA. Part B, Resources*, *2*(2), 804–805. https://doi.org/10.1080/23802359.2017.1398599
