# Supplementary material for: New Molecular Phylogenetic Evidence Confirms Independent Origin of Coxal Combs in the Families of the ‘Cydnoid’ Complex (Hemiptera: Heteroptera: Pentatomoidea)
Source: Insects. 2024 Oct 11;15(10):792. doi: 10.3390/insects15100792 (PMC11509079; doi:10.3390/insects15100792)
Supplement: Supplementary file 1 [file insects-15-00792-s001.zip › Table S2.pdf]

Table S2. List of specimens used for extraction and amplification during the present study. Their geographic origin, GenBank accession numbers, and University of Opole sample numbers are given. All newly sequenced specimens were identified to species by the first author (JAL).

Family	Subfamily	Tribe	Species	Geographic origin (year collected, if originally provided)	GenBank accesion numbers for the 16S rDNA sequences	University of Opole sample numbers for newly sequenced species
Cydnidae	Amaurocorinae	-	*Amaurocoris curtus* (Brullé, 1838)	Cyprus (2015)	PP357100	UO_226
	Amnestinae	-	*Amnestus ficus* Mayorga & Cervantes, 2001	Mexico (2014)	PP357101	UO_192Af1
			Amnestus pusio (Stål, 1860)	Guatemala (2011)	PP357102	UO_153Ap
			Amnestus zacki Mayorga & Cervantes, 2009	Guatemala (2010)	PP357103	UO_134Az
			Lattinestus amplus Eger, 2008	Costa Rica (1995)	PP357122	UO_29La
			Lattinestus barrerai Mayorga & Brailovsky, 2012	Guatemala (2007)	PP357123	UO_233Lb
	Cephalocteinae	Scaptocorini	*Stibaropus indonesicus* J.A. Lis, 1991	Brunei (2014)	PP357145	UO_218Ssp
			Stibaropus molginus (Schiødte, 1848)	Thailand (1995)	PP357146	UO_35Sm
	Garsauriinae	-	*Blaena setosa* Walker, 1868	Australia (2012)	PP357104	UO_179Bs
			Garsauria aradoides Walker, 1868	Brunei (2014)	PP357117	UO_217Ga
			Peltoxys sataranus J. A. Lis & B. Lis, 2007	India (2005)	PP357140	UO_32Psp
	Cydninae	Cydnini	*Chilocoris confusus* Horváth, 1919	Japan (2011)	PP357109	UO_166A
			Chilocoris neozealandicus Lariviére & Froeschner, 1994	Australia (1990)	PP357110	UO_24Cn
			Chilocoris capensis J.A. Lis, B. Lis & Compton, 2016	South Africa (2013)	PP357111	UO_194ChF1
			Cydnus aterrimus (Forster, 1771)	Poland (2010)	PP357113	UO_126A
			Nishadana umbrosa Horváth, 1919	India (2005)	PP357131	UO_23Nu
			Parachilocoris minutus (Distant, 1901)	Japan (2011)	PP357139	UO_168
		Geotomini	*Adrisa birmana* J.A. Lis, 1992	Thailand (2013)	PP357095	UO_181Asp1
			Adrisa magna (Uhler, 1861)	Japan (2010)	PP357096	UO_143A
			Adrisa romani J.A. Lis, 1994	Thailand (2013)	PP357097	UO_182Asp2
			Aethus pseudindicus J.A. Lis, 1993	Vietnam (2013)	PP357098	UO_7Ap
			Alonips obsoletus Signoret, 1881	Australia (2018)	PP357099	UO_13Ao
			Byrsinus pseudosyriacus (Linnavuori, 1977)	Namibia (2012)	PP357105	UO_22Bp
			Byrsinus varians (Fabricius, 1803)	Guam (2004)	PP357106	UO_149Bv
			Cyrtomenus emarginatus Stål, 1862	Guatemala (2011)	PP357114	UO_152Ce
			Fromundus difficilis (Stål, 1854)	Namibia (2012)	PP357115	UO_175
			Fromundus pygmaeus (Dallas, 1851)	Guam (2004)	PP357116	UO_151C
			Geotomus convexus Hsiao, 1977	Japan (2011)	PP357118	UO_167
			Katakadia caliginosa (Walker, 1867)	Brunei (2014)	PP357119	UO_221Kc

			Lactistes obesipes Signoret, 1879	Australia (2018)	PP357120	UO_14Lo
			Lactistes vericulatus Schiødte, 1848	Zambia (2014)	PP357121	UO_15Lv
			Macroscytus annulipoides J.A. Lis, 1999	Australia (2018)	PP357125	UO_190519_16Ma
			Macroscytus badius (Walker, 1867)	India (2013)	PP357126	UO_1Mb
			Macroscytus fraterculus Horváth, 1919	Japan (2010)	PP357127	UO_142
			Macroscytus japonensis Scott, 1874	Vietnam (2013)	PP357128	UO_190519_4M
			Macroscytus minimus J.A. Lis, 1999	Australia (2018)	PP357129	UO_190519_12Mm
			Microporus pallidipennis (Reuter, 1883)	Namibia (2012)	PP357130	UO_172A
			Pangaeus bilineatus (Say, 1825)	Guatemala (2011)	PP357137	UO_170A
			Pangaeus rugiceps Horváth, 1919	Guatemala (2011)	PP357138	UO_171Pr
			Pseudoscoparipes fraterculus J.A. Lis, 1994	Thailand (2013)	PP357141	UO_20Pb
			Pseudoscoparipes kinabalensis J.A. Lis, 1994	Brunei (2015)	PP357142	UO_219Pk (CD)
			Pseudoscoparipes vollenhoveni (Signoret, 1881)	Brunei (2015)	PP357143	UO_190519_18Pv
			Rhytidoporus indentatus Uhler, 1877	Guam (2004)	PP357144	UO_150A
			Teabooma secunda J.A. Lis et B. Lis, 2010	New Caledonia (2004)	PP357147	UO_19Tp
	Sehirinae	Sehirini	*Adomerus biguttatus* (Linnaeus, 1758)	Poland (2010)	PP357091	UO_127A
			Adomerus rotundus Hsiao, 1977	Japan (2010)	PP357092	UO_138A
			Adomerus triguttulus (Motschulsky, 1866)	Japan (2010)	PP357093	UO_139A
			Adomerus variegatus (Signoret, 1884)	Japan (2010)	PP357094	UO_140A
			Canthophorus impressus Horváth, 1881)	Poland (2010)	PP357107	UO_128A
			Canthophorus niveimarginatus Scott, 1874	Japan (2010)	PP357108	UO_141A
			Crocistethus waltlianus (Fieber, 1837)	Morocco (2002)	PP357112	UO_230
			Legnotus limbosus (Geoffroy, 1785)	Poland (2014)	PP357124	UO_187Ll
			Ochetostethomorpha secunda J.A. Lis & B. Lis, 2014	Namibia (2012)	PP357132	UO_173
			Ochetostethus brachyscytus Reuter, 1891	Egypt (1997)	PP357133	UO_49Ob
			Ochetostethus heissi Magnien, 2006	Cyprus (2015)	PP357134	UO_36Osp
			Ochetostethus nanus Herrich-Schaeffer, 1834	Spain (1988)	PP357135	UO_233Oo
			Ochetostethus opacus (Scholtz, 1847)	Poland (2016)	PP357136	UO_223Oo
			Tritomegas bicolor (Linnaeus, 1758)	Poland (2011)	PP357148	UO_144
			Tritomegas sexmaculatus (Rambur, 1839)	Poland (2010)	PP357149	UO_1D1
Nabidae	Nabinae	Nabini	*Himacerus mirmicoides* (O. G. Costa, 1834)	Poland (2010)	PP357152	UO_T40
Pentatomidae	Podopinae	Graphosomatini	*Graphosoma italicum* (O.F. Müller, 1766)	Czech Republic (2011)	PP357151	UO_157C
Thyreocoridae	Corimelaeninae	-	*Galgupha australis* McAtee & Malloch, 1933	Bolivia (2010)	PP357150	UO_163A
