# Supplementary figures and images for: New Molecular Phylogenetic Evidence Confirms Independent Origin of Coxal Combs in the Families of the ‘Cydnoid’ Complex (Hemiptera: Heteroptera: Pentatomoidea)
Source: Insects. 2024 Oct 11;15(10):792. doi: 10.3390/insects15100792 (PMC11509079; doi:10.3390/insects15100792)

ML

BI

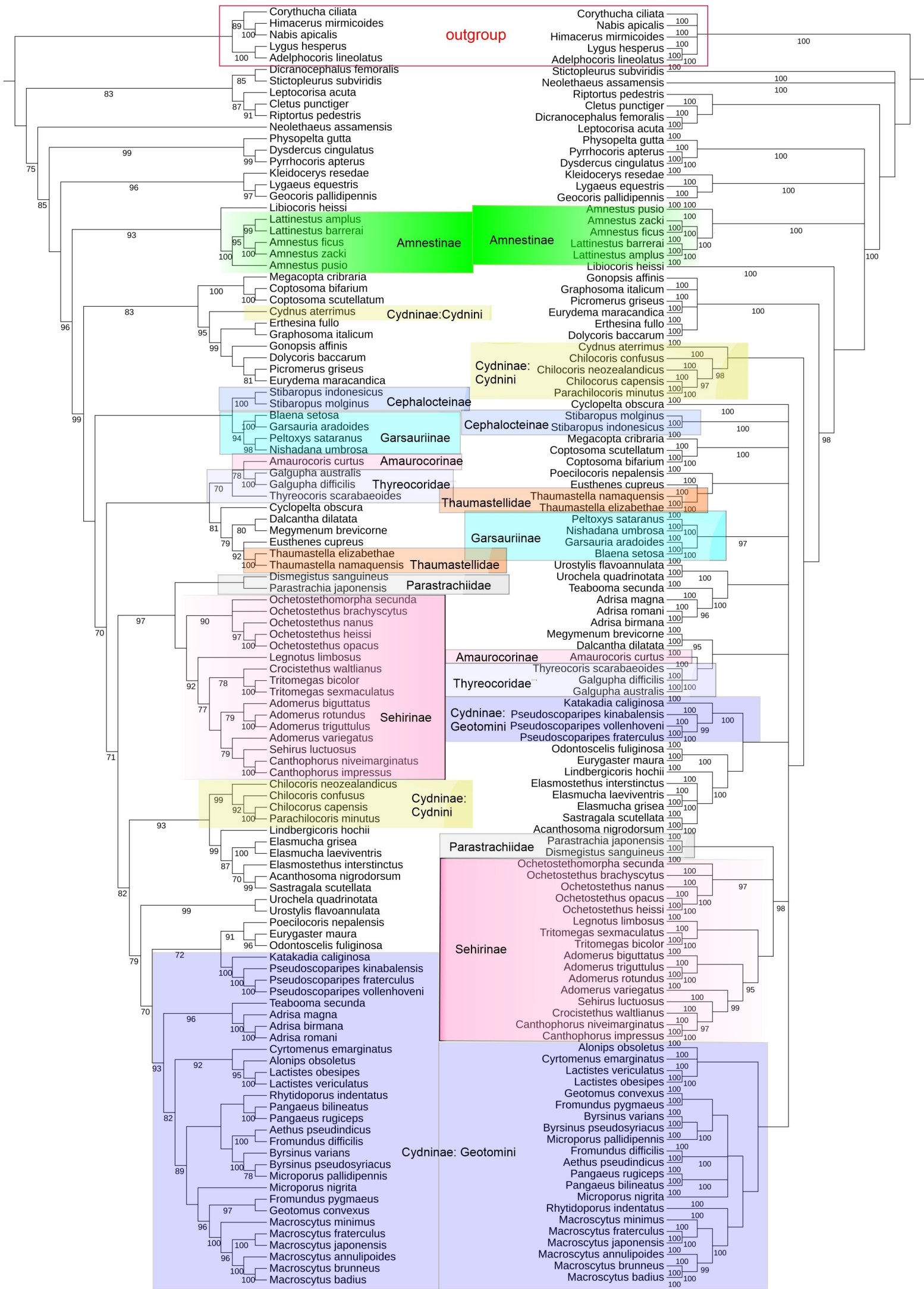

Supplement: Supplementary file 1 [file insects-15-00792-s001.zip › Figure S1.pdf]
